# Dynamic IL-6R/STAT3 signaling leads to heterogeneity of metabolic phenotype in pancreatic ductal adenocarcinoma cells

**DOI:** 10.1016/j.celrep.2023.113612

**Published:** 2023-12-23

**Authors:** Wiktoria Blaszczak, Bobby White, Stefania Monterisi, Pawel Swietach

**Affiliations:** 1 Department of Physiology, Anatomy & Genetics, University of Oxford, Sherrington Building, Parks Road, OX1 3PT Oxford, UK

**Keywords:** variation, homeostasis, fermentation, glycolysis, PDAC, feedback, interleukin-6, SOCS, microenvironment, heterogeneity, rationing

## Abstract

Malignancy is enabled by pro-growth mutations and adequate energy provision. However, global metabolic activation would be self-terminating if it depleted tumor resources. Cancer cells could avoid this by rationing resources, e.g., dynamically switching between “baseline” and “activated” metabolic states. Using single-cell metabolic phenotyping of pancreatic ductal adenocarcinoma cells, we identify MIA-PaCa-2 as having broad heterogeneity of fermentative metabolism. Sorting by a readout of lactic acid permeability separates cells by fermentative and respiratory rates. Contrasting phenotypes persist for 4 days and are unrelated to cell cycling or glycolytic/respiratory gene expression; however, transcriptomics links metabolically active cells with interleukin-6 receptor (IL-6R)-STAT3 signaling. We verify this by IL-6R/STAT3 knockdowns and sorting by IL-6R status. IL-6R/STAT3 activates fermentation and transcription of its inhibitor, SOCS3, resulting in delayed negative feedback that underpins transitions between metabolic states. Among cells manifesting wide metabolic heterogeneity, dynamic IL-6R/STAT3 signaling may allow cell cohorts to take turns in progressing energy-intense processes without depleting shared resources.

## Highlights

• PDAC cells sorted by lactic acid efflux capacity yield metabolically distinct sub-populations
• The contrast in fermentative rate is short lived and linked to IL-6R/STAT3 signaling
• IL-6R/STAT3 activation is transient under delayed negative feedback from SOCS3
• The ensuing metabolic heterogeneity may facilitate rationing of tumor resources

## Graphical abstract

**Figure:**
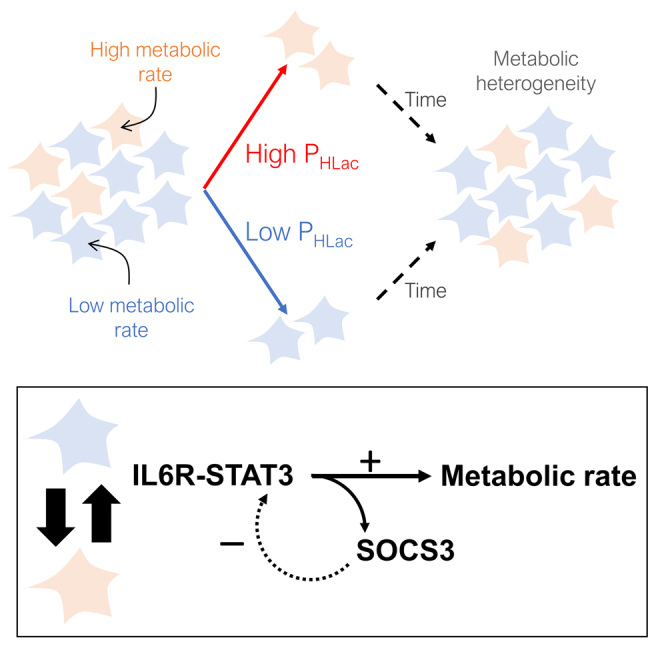

## Teaser

Blaszczak et al. describe a mechanism for metabolic heterogeneity in PDAC cells, driven by transient activation of the IL-6R/STAT3 pathway. To separate cells by fermentative rate, the authors design a sorting protocol that sortscells by lactic acid efflux capacity and allows phenotyping of the emergent sub-populations.

## Introduction

Mutations enabling rapid proliferation are selected positively in cancers because they accelerate the emergence of aggressive phenotypes through somatic evolution.^1^^,^^2^ However, proliferation is costly in terms of energy and substrates, and maintaining a persistently raised metabolic rate would be counter-productive if it caused the tumor to become depleted of resources and overloaded with waste.^3^ One way of managing resources is through rationing, which could be implemented by alternating the metabolic activity of cells between “low” and “high” states, thereby establishing dynamic heterogeneity. Intriguingly, a recent study determined that “bulk” fermentative and respiratory rates in primary tumors are lower than previously postulated,^4^ which argues against population-wide metabolic hyper-activation but leaves open the possibility of sub-populations with transiently higher metabolic rates. Indeed, single-cell methods applied to cancer cells have described heterogeneity in terms of transcriptomics, epigenomics, metabolomics, and proteomics,^5^^,^^6^ which can impact metabolic rate.^6^^,^^7^ However, most observations have been based on “snapshots” that describe heterogeneity at the point of measurement but cannot predict dynamics over time. It is therefore unclear whether reported metabolic heterogeneity is a manifestation of persistent differences between cells or of fluctuating activities across the population. This distinction is critical because genotypes with hard-wired metabolic activation may have an inherent growth advantage and could, with time, overtake the tumor. A sustainable form of dynamic heterogeneity would require cells to seamlessly alternate between metabolic states. Such a system could be implemented by coupling metabolism onto signaling cascades that show rhythm. One example is the cell cycle,^8^ but this arrangement would constrain metabolic activation to specific stages of the cell cycle, which may not necessarily deliver the best matching between metabolic demand and supply, particularly during longer phases of the cycle. Moreover, it could be argued that metabolic activation should instruct cell-cycle progression, rather than vice versa, to ensure optimal use of resources.

Here, we sought evidence and a mechanism for dynamic metabolic heterogeneity in cancer cell lines. Pancreatic ductal adenocarcinoma (PDAC) lines were chosen for our study because cancers of the pancreas are characterized by rapid progression,^9^^,^^10^ invasiveness,^11^^,^^12^ and elevated fermentative metabolism.^13^^,^^14^^,^^15^^,^^16^ Their high “bulk” glycolytic rate has been linked to mutations; for example *KRAS* mutations lead to sustained activation of MAPK and PI3K-mTOR pathways^16^^,^^17^ and mutations in *TP53* disinhibit glucose metabolism.^18^^,^^19^ However, the extent and causes of within-population metabolic heterogeneity remain unclear. We speculate that metabolic heterogeneity can arise when upstream regulators manifest rhythmicity, such as that produced by delayed negative feedback.^20^ The time delay in this circuit could be regulated to optimize periodicity for tumor growth. An analogy can be drawn to circadian rhythms,^21^ yet mechanisms relevant to cancer metabolism are unclear.

A challenge to studying metabolic heterogeneity is identifying a readout that accurately distinguishes cells by metabolic rate. Since metabolism is an ensemble process that depends on a network of proteins and their kinetic and thermodynamic constraints, single-cell methods that measure gene expression may not adequately separate sub-populations by metabolic flux. A recent study^7^ took a leap forward by studying metabolic heterogeneity in terms of glucose levels imaged using a fluorescent sensor. A limitation of this approach is that steady-state concentrations cannot predict metabolite fluxes. Although glucose uptake is the first step in metabolism, its relationship to metabolic flux is less intuitive. For example, it is unclear whether a cell with high glucose content is metabolically inactive because glucose is allowed to build up in the cytoplasm, or active because it requires a high rate of glucose uptake. Moreover, that study also determined glucose levels to be heritable, a trait that may cause phenotypic drift under selection pressures, and thus less likely to preserve metabolic heterogeneity in the long term. For the same reasoning, single-point measurements of the abundance of metabolic intermediates cannot quantify flux. An additional concern with sorting strategies based on metabolite concentrations is that these are vulnerable to stresses imposed by the experimental method. For example, preparation and processing for fluorescence-activated cell sorting (FACS) may acutely change the metabolic activity of cells.

To address these concerns, we propose a new strategy for separating cells by metabolic activity that is based on the cell's capacity to remove lactic acid, measured in terms of permeability (P_HLac_). We reasoned that cells with high fermentation rates require adequate capacity to conduct an efflux of lactate across the membrane to match their glycolytic production. Cells with the highest P_HLac_ can be identified flow cytometrically from the large intracellular alkalinization that is triggered when lactate is rapidly driven out of cells down an experimentally controlled gradient. Among the PDAC lines, we identified MIA PaCa-2 as having the broadest metabolic heterogeneity and used these cells to validate our sorting strategy. We analyzed the emergent sub-populations to study the mechanisms that produce metabolic contrasts. Our results identify the interleukin-6 receptor (IL-6R)/STAT3 signaling pathway as a delayed negative feedback circuit that shows rhythmic activity^21^ and drives changes in metabolic rate. We propose that alternating between low and high metabolic states allows cancer cells to randomize commitments to resource-intense events (e.g., protein synthesis),^4^^,^^6^ and protect their shared resources, even when programmed genetically to hyper-proliferate.

## Results

### Heterogeneity in fermentative rate varies between PDAC cell lines

**Figure 1:**
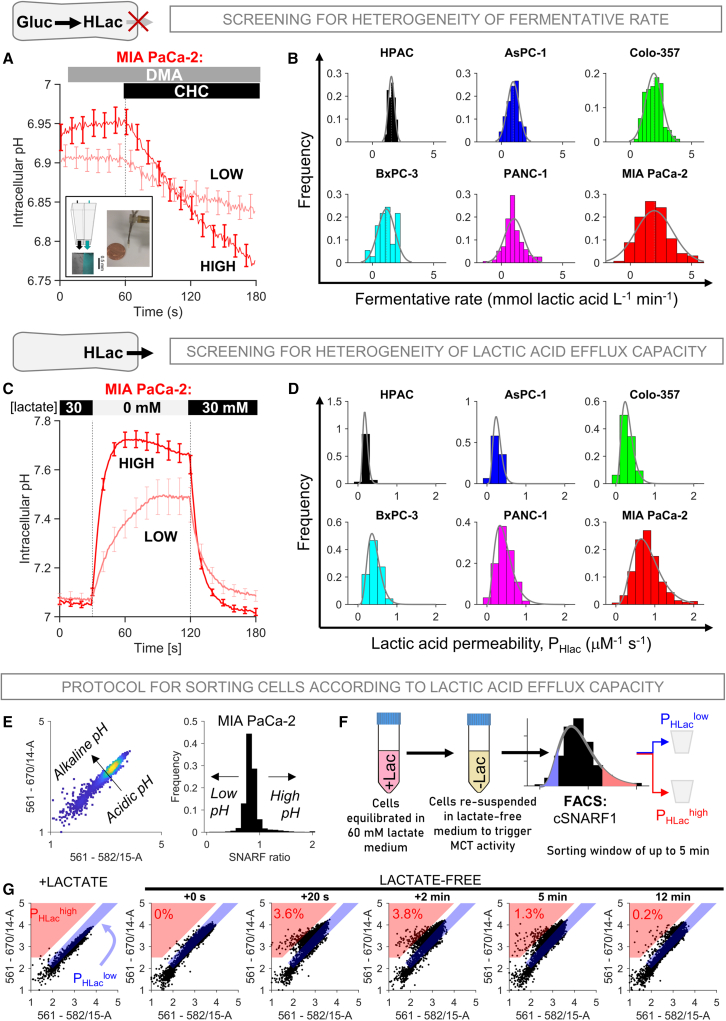
Heterogeneity of fermentative metabolism in PDAC cells (A) Measuring fermentation rate from the buildup of cytoplasmic acid upon rapid MCT inhibition with CHC (2 mM); inset shows a rapid-switcher device. pHi was measured in cSNARF1-loaded MIA PaCa-2 cells superfused with CO_2_/HCO_3_^−^-free buffer (blocks HCO_3_^−^ transport) containing 5-*N*,*N*-dimethyl-amiloride (DMA; 30 μM; blocks Na^+^/H^+^ exchange). Time courses show averaged recordings from cells in the top 20% or bottom 20% fermentative rate (∼300 cells/five biological repeats). Mean ± SEM. (B) Frequency histogram (best-fit Gaussian distribution) of fermentation rate, ranked by increasing variance (n = 31, 48, 117, 37, 157, and 299) from at least 3 biological repeats. (C) Measuring membrane lactic acid permeability (P_HLac_). Time course of pHi in cSNARF1-loaded MIA PaCa-2 cells representing the highest 15% and lowest 15% P_HLac_ (∼300 cells/five biological repeats) is shown. Mean ± SEM. (D) Frequency histogram (best-fit gamma distribution) for P_HLac_ ranked by increasing variance (n = 52, 138, 128, 196, 187, and 304) from at least 4 biological repeats. (E) Steady-state pHi (cSNARF1 ratio). (F) Protocol for sorting cells by P_HLac_. Faster and larger pHi transients separate P_HLac_^high^ cells (red) from P_HLac_^low^ cells (blue). Exemplar recordings show the emergence of the P_HLac_^high^ population in the red-shaded region.

We implemented a single-cell fluorimetric assay to quantify metabolic heterogeneity in a panel of PDAC cell lines spanning a range of bulk fermentative rates.^22^ Fermentation generates lactic acid, which must exit across the membrane via monocarboxylate transporters (MCTs) as H^+^-lactate and, to a lesser degree, through the lipid matrix as undissociated lactic acid. Rapid MCT inhibition leads to an abrupt cytoplasmic buildup of lactate and H^+^ ions, the latter of which can be measured using pH indicators, such as 5-(and-6)-Carboxy SNARF-1 (cSNARF1), loaded into cells. Consequently, the rate of intracellular acidification is a single-cell readout of fermentative rate.^22^ To prevent regulators of intracellular pH (pHi) from attenuating this response, CO_2_/HCO_3_-free buffer was used to inactivate HCO_3_^−^-dependent transporters, and 5-*N,N*-dimethyl-amiloride (DMA) was included to inhibit Na^+^/H^+^ exchange, the major transporter remaining in CO_2_/HCO_3_^−^-free conditions. A dual microperfusion device rapidly switched between two microstreams, one of which contained 2 mM α-cyano-4-hydroxycinnamate (CHC) to block MCTs^23^ and trigger immediate acidification at a rate equal to lactic acid production (Figure 1A). Repeated measurements described the statistical distribution of fermentative rate, which was narrowest in HPAC and broadest in MIA PaCa-2 (Figure 1B). Cell line authentication (Table S1) excluded contamination as a potential cause of variation.

### Lactic acid permeability as a sorting strategy for studying metabolic heterogeneity

To study the mechanisms of metabolic heterogeneity, we sought ways of separating cells using FACS-compatible readouts. We first tested uptake of a fluorescent non-metabolizable glucose derivative, 2-NBDG (2-deoxy-2-[(7-nitro-2,1,3-benzoxadiazol-4-yl) amino]-D-glucose). After wash-out, MIA PaCa-2 cells were sorted into low- and high-fluorescence groups (Figure S1A). The two sub-populations were cultured for 24 h and assayed for fermentative rate using acidification of lightly buffered medium as a direct readout of lactic acid production.^24^ Strikingly, the difference in fermentative rate between 2-NBDG-high and 2-NBDG-low cells did not reach statistical significance, indicating that this sorting strategy is inadequate for studying heterogeneity (Figures S1B, NB: 2-NBDG loading did not inhibit glycolysis, and S1C). Next, we considered a genetically encoded lactate sensor, Laconic.^25^ Although Laconic detected a rise in [lactate] when superfusates were switched from galactose containing to lactate containing, the response was modest and comparable to baseline variation (Figure S1D). This signal-to-noise ratio is inadequate to accurately separate cells by metabolic status.

Recognizing that point-measurements of metabolite concentrations cannot infer flux, we sought an alternative sorting strategy using a parameter that is more closely related to metabolic rate. Under the constraints of flux balance, cells with a higher glycolytic rate are expected to have greater capacity to remove lactic acid across the membrane. The latter was interrogated using a validated method^22^ that recorded the pHi response to switching between a microstream containing 30 mM lactate and one that was lactate free (Figure 1C). The rate of pHi change, after factoring buffering capacity, measures the membrane’s permeability to lactic acid (P_HLac_).^22^ The P_HLac_ distribution was narrowest in HPAC and broadest in MIA PaCa-2 (Figure 1D), matching the distributions of fermentative rate (Figure 1B). To test if lactic acid permeability correlates with fermentative rate, we obtained sub-populations sorted by P_HLac_ for further measurements. Cells from the top and bottom deciles of the P_HLac_ distribution produced lactate-evoked pHi transients of markedly different time courses, providing a window for flow-cytometric sorting using pHi-sensitive fluorescence (Figure 1E). To implement this, cSNARF1-loaded cells were equilibrated with 60 mM lactate and then transferred to lactate-free medium immediately prior to cell sorting (Figure 1F). Cells with the highest P_HLac_ (P_HLac_^high^) produced the most prominent alkaline transients, distinguishing them from P_HLac_^low^ cells (Figure 1G).

### Sorting by P_HLac_ produces contrasting but short-lived metabolic phenotypes

**Figure 2:**
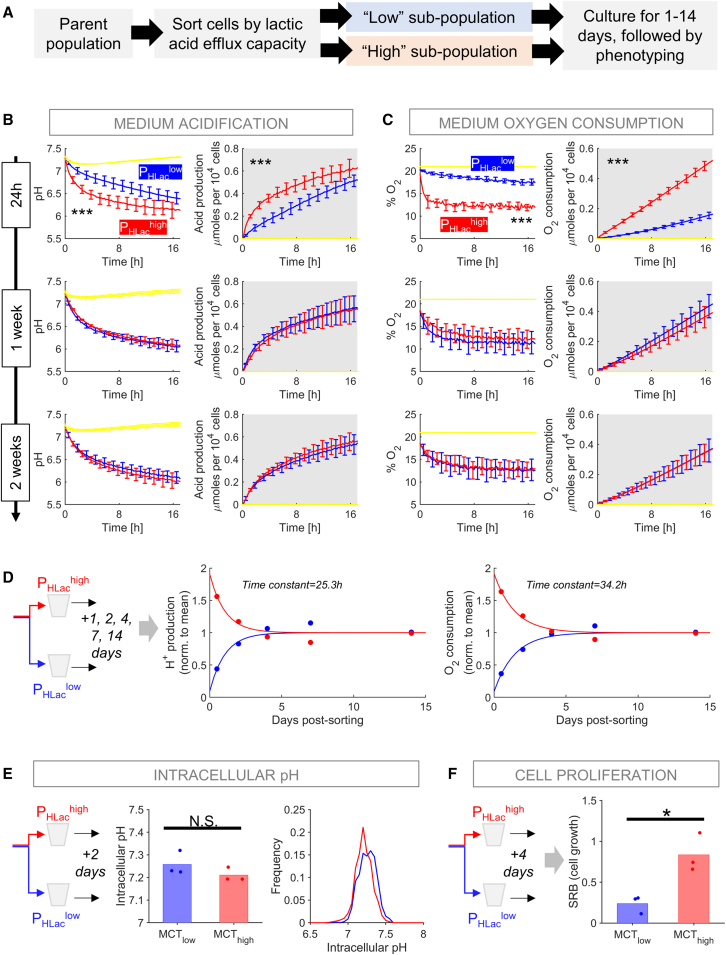
Phenotyping sub-populations gated by P_HLac_ (A) Workflow for phenotyping MIA PaCa-2 sub-populations obtained by sorting for P_HLac_. (B) Fluorimetric assay of fermentation rate in P_HLac_^high^ and P_HLac_^low^ sub-populations, measured after 1, 7, or 14 days in culture post-sorting (n = 12/N = 4 for each). Medium pH and cumulative acid production were calculated from pH time course. Significant difference (p < 0.001) between P_HLac_^high^/P_HLac_^low^ after 1 day of sorting (two-way ANOVA). Mean ± SEM. (C) Medium dissolved O_2_ concentration (%) and cumulative oxygen consumption calculated from O_2_ time course. Mean ± SEM. (D) Analysis of the initial rate of H^+^ production and O_2_ consumption in P_HLac_^low^ and P_HLac_^high^ sub-populations at various days post-sorting, fitted to mono-exponential function. (E) Intracellular pH measured in P_HLac_^low^ and P_HLac_^high^ cells after 2 days of culture post-sorting; no significant difference (t test; N = 3 independent sorts; n >1,000 cells per measurement). (F) Cell biomass (SRB assay) performed after 4 days of culture of P_HLac_^low^ and P_HLac_^high^ sub-populations. Nested t test: p = 0.0164 (N = 3 independent sorts, each with 5–8 technical repeats).

P_HLac_-sorted sub-populations were profiled by real-time fluorimetric measurements of medium acidification (fermentative rate) and O_2_ consumption (respiratory rate)^24^ (Figure 2A). After 24 h culture, fermentative and respiratory rates were, respectively, 3- and 4-fold higher in P_HLac_^high^ cells after controlling for cell density (Figures 2B and 2C). Thus, the cell’s ability to conduct an efflux of lactic acid is a strong predictor of fermentative as well as respiratory rate. To study the longevity of these contrasting metabolic phenotypes, sorted P_HLac_ sub-populations were cultured for up to 14 days prior to phenotyping. The metabolic contrast between P_HLac_^low^ and P_HLac_^high^ cells decreased with a time constant of ∼30 h and collapsed by day 4 (Figures S2 and 2D). Since a minimum of 24 h is needed for sorted cells to settle on plates for assays, it is not possible to characterize the metabolic contrast immediately after sorting. However, extrapolating the time courses in Figure 2D to the point of sorting indicates that the initial metabolic contrast may be an order of magnitude. Although the sorting process was based on measuring pHi responses to a lactate maneuver, the emergent sub-population did not differ in resting pHi (Figure 2E). This means that the metabolic contrast cannot be explained in terms of steady-state pHi or differences in pHi regulators.

The short-lived nature of the metabolic contrast argues against an underlying heritable factor. Population-level heterogeneity in P_HLac_ was also unrelated to cell-cycle stage, as determined in experiments using GFP-tagged geminin transfected into MIA PaCa-2 cells and nuclear Hoechst fluorescence intensity (Figure S3A). Moreover, sorting by P_HLac_ was still able to separate cells by fermentative rate after cell-cycle synchronization with hydroxyurea, which arrests cells in G1/S phase and prevents entry to G_2_ (Figures S3B and S3D). These findings suggest that metabolic differences are not strictly coupled to specific phases of the cell cycle.

The higher metabolic rate in P_HLac_^high^ cells is expected to facilitate energetically expensive processes, such as growth of cellular biomass. This was tested by sulforhodamine B (SRB) assay of cells cultured post-sorting. Longer growth periods improve the resolving power to seek differences in biomass, but since the metabolic contrast between sub-populations collapsed by day 4, the time frame for measuring cell growth was 4 days post-sorting. We found that P_HLac_^high^ cells grew 4-fold faster than P_HLac_^low^ cells, consistent with a matching between metabolic activity and resource-intense biological processes (Figure 2F). However, the observation that P_HLac_-sorted sub-populations returned symmetrically to a mid-point metabolic phenotype within 4 days indicates that any growth-accelerating trait in P_HLac_^high^ cells is transient; otherwise, these cells would have dominated the population. Thus, metabolic heterogeneity in MIA PaCa-2 cells appears to arise from a dynamic equilibrium between cells of high and low metabolic rates.

### Sorting cells by P_HLac_ reveals transcriptionally distinct sub-populations

**Figure 3:**
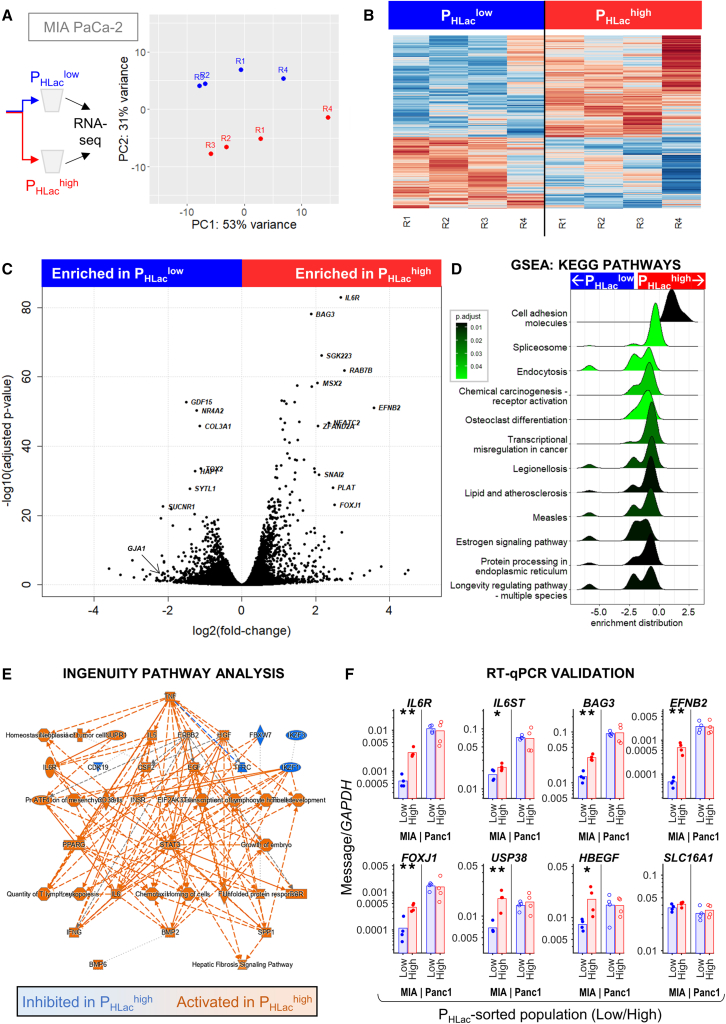
Gene expression analysis of sub-populations sorted by P_HLac_ (A) Principal component analysis of samples prepared from four independent sorts. (B) Heatmap showing differentially expressed genes (DEGs): 1,510 higher in P_HLac_^low^ cells and 2,156 higher in P_HLac_^high^ cells (cutoff: adjusted p < 0.05). (C) Volcano plot showing DEGs. Highlighted upregulated DEGs: log2(fold change) > 2, p.adj < 10^−25^; highlighted downregulated DEGs: log2(fold change) < −1, p.adj < 10^−25^. (D) KEGG pathway analysis by gene-set enrichment analysis (GSEA) performed by ClusterProfiler: minimum/maximum gene set size 20/1,000, p < 0.05. Ridge plot shows distribution of DEGs grouped by gene set: x axis shows log fold change (LFC), color denotes false discovery rate-adjusted significance, and shape shows the frequency distribution of DEGs by their LFC. (E) Results of hierarchical Ingenuity Pathway Analysis (IPA). (F) RT-qPCR confirmation of selected genes, normalized to GAPDH. Data are for MIA PaCa-2 (labeled as MIA; filled symbols) and PANC-1 cells (empty symbols).

We performed RNA sequencing (RNA-seq) to investigate the underlying causes of the metabolic difference between P_HLac_-sorted sub-populations. Experiments were performed on pairs of samples from four independent flow cytometric sorts, and the RNA was extracted within 2 h post-sorting. P_HLac_^high^ and P_HLac_^low^ sub-populations resolved well on principal-component analysis (Figure 3A) and showed distinct patterns of gene expression (Figure 3B; Table S2). Analysis (DESeq2) identified differentially expressed genes (DEGs; adjusted p value [p.adj] < 0.05) enriched in P_HLac_^high^ (2,156 genes) and P_HLac_^low^ cells (1,510 genes; Figure 3C). Gene set enrichment analysis (clusterProfiler) identified several KEGG pathways enriched among DEGs; the most prominently upregulated pathway in P_HLac_^high^ cells related to cell adhesion (Figures 3D and S4). Consistent with our earlier findings, these pathways did not include the cell cycle. Strikingly, canonical glycolytic or respiratory pathways were not enriched in P_HLac_^high^ cells (“glycolysis/gluconeogenesis,” p.adj = 0.79; “oxidative phosphorylation,” p.adj = 0.79). This finding indicates that the phenotypic differences between P_HLac_^high^ and P_HLac_^low^ cells could not have been predicted from transcripts related to fermentation and respiration. Instead, phenotype may be shaped by the state of signaling and contributions from thermodynamic, allosteric, and post-translational factors that are not captured by transcriptomics. Additionally, neither population was enriched in PDAC stemness markers (Figures S4E and S4F).

To identify signaling pathways enriched in P_HLac_^high^ and P_HLac_^low^ cells, we performed hierarchical Ingenuity Pathway Analysis. This highlighted the IL-6R/STAT3 pathway as prominently upregulated in P_HLac_^high^ cells (Figure 3E), which is consistent with the role of IL-6 in stimulating metabolism.^26^^,^^27^^,^^28^^,^^29^ Among the DEGs, we identified a strong upregulation of the *IL6R* alpha receptor and its dimerization partner *IL6ST*. Selected DEGs were verified by RT-qPCR analysis of independently collected MIA PaCa-2 samples (Figure 3F). Strikingly, the gene coding for MCT1, *SLC16A1* (the activity of which is used for sorting experiments), was not differentially expressed between P_HLac_^high^ and P_HLac_^low^ cells, which exemplifies the importance of sorting according to functional readouts rather than transcript levels of specific genes or their protein products. In agreement with the short-lived contrast between metabolic phenotypes, the transcriptional difference between P_HLac_^low^ and P_HLac_^high^ sub-populations, in terms of selected DEGs, collapsed after 10 days of culture post-sorting (Figure S5).

The expression of DEGs associated with metabolic heterogeneity in MIA PaCa-2 cells was compared across PDAC lines using bulk RNA-seq datasets available from the Cancer Cell Line Encyclopedia (CCLE).^30^ Genes enriched in P_HLac_^high^ cells tended to have low bulk expression levels in MIA PaCa-2 cells compared to other PDAC lines (Figures S6A and S6B). This suggests that metabolically active MIA PaCa-2 cells may represent a small subset of the entire population, which otherwise expresses low levels of the highlighted DEGs. Next, each DEG identified between P_HLac_^low^ and P_HLac_^high^ MIA PaCa-2 cells was assigned a correlation coefficient that described its trend in the ranking of lines by ascending metabolic heterogeneity (AsPC-1 < BxPC-3 < PANC-1 < MIA PaCa-2). This analysis showed that the most prominent DEGs were assigned a negative correlation coefficient, i.e., had the lowest bulk expression in MIA PaCa-2 cells (Figure S6C). Moreover, “positive regulation of tyrosine phosphorylation of STAT protein” (but not glycolysis or respiration ontologies) had the lowest bulk expression in MIA PaCa-2 (Figure S6D).

We speculate that MIA PaCa-2 cells may support heterogeneity by maintaining bulk transcript levels of DEGs at a low level, but allowing transient induction in some cells, producing higher metabolic rate. To investigate this, RT-qPCR measurements were performed on PANC-1 cells, which have a high bulk fermentation rate but narrower metabolic heterogeneity compared to the MIA PaCa-2 line (Figure 1A). In contrast to the stark transcriptional contrasts in MIA PaCa-2 cells, PANC-1 sub-populations sorted by P_HLac_ showed small and non-significant differences in gene expression of these genes (Figure 3F). Moreover, *IL6R*, *IL6ST*, BAG4, *EFNB2*, and *FOXJ1* were more highly expressed in PANC-1 compared to MIA PaCa-2 cells, consistent with the CCLE analyses. A saturation phenomenon may limit the ability of PANC-1 cells to generate heterogeneity.

### Metabolic heterogeneity is related to the state of IL-6R/STAT3 signaling

**Figure 4:**
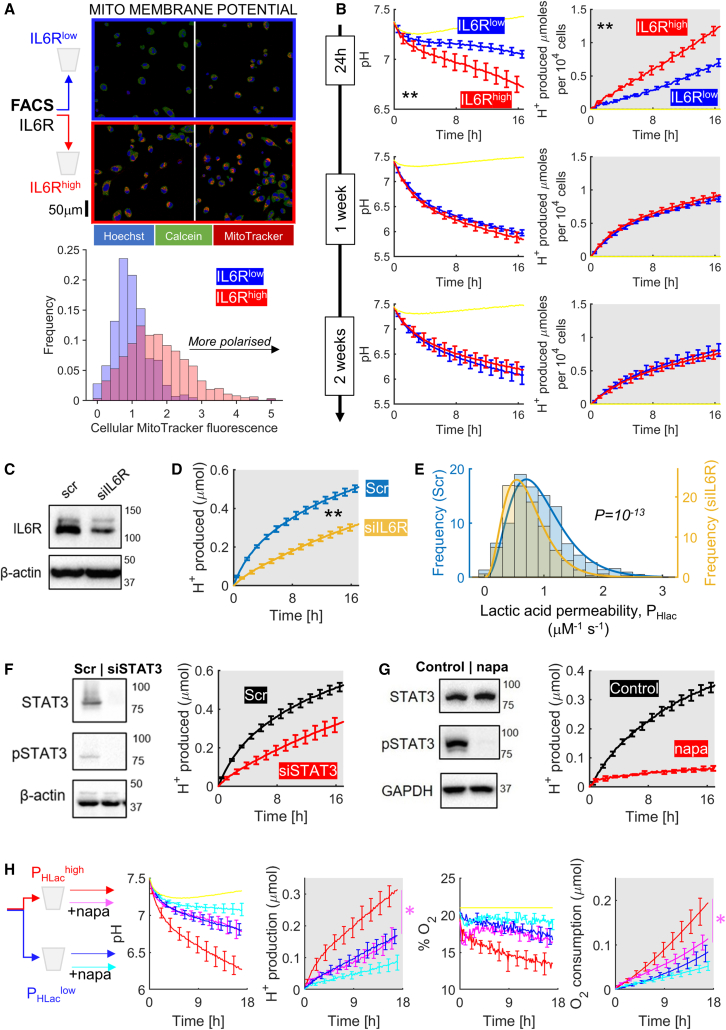
Linking metabolic heterogeneity to the IL-6-STAT3 pathway (A) MIA PaCa-2 cells sorted by IL-6R status to separate top and bottom 15% of cells. Sub-populations were plated for live-cell imaging using MitoTracker Red. Calcein was used to stain cytoplasmic areas and Hoechst identified nuclei. Histogram shows results from three independent experiments, normalized to IL-6R^low^. (B) Cells sorted by IL-6R status were plated for fluorimetric assays of fermentation rate after 1, 7, and 14 days in culture (N = 5). Mean ± SEM. (C) Western blot confirmation of *IL6R* knockdown. (D) *IL6R* knockdown reduced fermentation rate (N = 5). Mean ± SEM. (E) *IL6R* knockdown reduced heterogeneity of P_HLac_ by ablating sub-population of highest P_HLac_ (N = 3; Kolmogorov-Smirnov test). (F) Western blot for STAT3 and its phosphorylated form, confirming *STAT3* knockdown. *STAT3* knockdown reduced the fermentation rate (N = 5). Mean ± SEM. (G) Western blot for STAT3 and its phosphorylated form, confirming inhibitory effect of napabucasin (1 μM). STAT3 inhibition reduced the fermentative rate (N = 5). Mean ± SEM. (H) Cells sorted by P_HLac_ and plated for fluorimetric phenotyping of fermentative and respiratory rate. Paired measurements, with and without napabucasin (napa; 1 μM) added to cells prior to measurements. Significant effect of napa in P_HLac_^high^ cells only (two-way ANOVA). Mean ± SEM.

Cells sorted by high P_HLac_ had a raised metabolic rate and *IL6R* expression, but this does not prove a causal link between IL-6R and metabolic activation. This was tested by sorting MIA PaCa-2 cells by IL-6R surface expression and measuring metabolic readouts of IL-6R^low^ and IL-6R^high^ sub-populations. After 24 h culture, IL-6R^high^ cells had more polarized mitochondria, imaged with MitoTracker (Figure 4A), and produced a higher fermentative rate (Figure 4B, NB: too few cells were collected to measure respiration). The metabolic contrast between IL-6R^low^ and IL-6R^high^ cells disappeared after a week of culture, in agreement with observations from P_HLac_-sorted cells. The independent observations that sorting by a metabolic proxy (P_HLac_) identified IL-6R positivity and sorting by IL-6R identified metabolically active cells confirms a causal relationship between IL-6R and metabolic heterogeneity. This link was verified by small interfering RNA (siRNA) knockdown (Figure 4C), which decreased population-averaged fermentative rate (Figure 4D) and the narrowed the P_HLac_ distribution (Figure 4E).

IL-6R evokes a myriad of responses, including metabolic actions, through STAT3.^31^^,^^32^^,^^33^^,^^34^ The role of STAT3 as an activator of fermentative metabolism was tested by STAT3 knockdown or inhibition (napabucasin). Knockdown ablated STAT3 expression, and acute treatment with napabucasin (1 μM) reduced STAT3 phosphorylation; both reduced fermentative rate (Figures 4F, 4G, and S7A). The rapid onset of the napabucasin effect suggests that STAT3 controls fermentation via a non-transcriptional mechanism. Strikingly, the effect of STAT3 inhibition on blocking fermentation transiently stimulated respiration, which may be a compensatory response (Figure S7B). Consistent with metabolically active cells being associated with IL-6R/STAT3 signaling, the metabolic response to napabucasin was greater in P_HLac_^high^ cells (Figure 4H).

**Figure 5:**
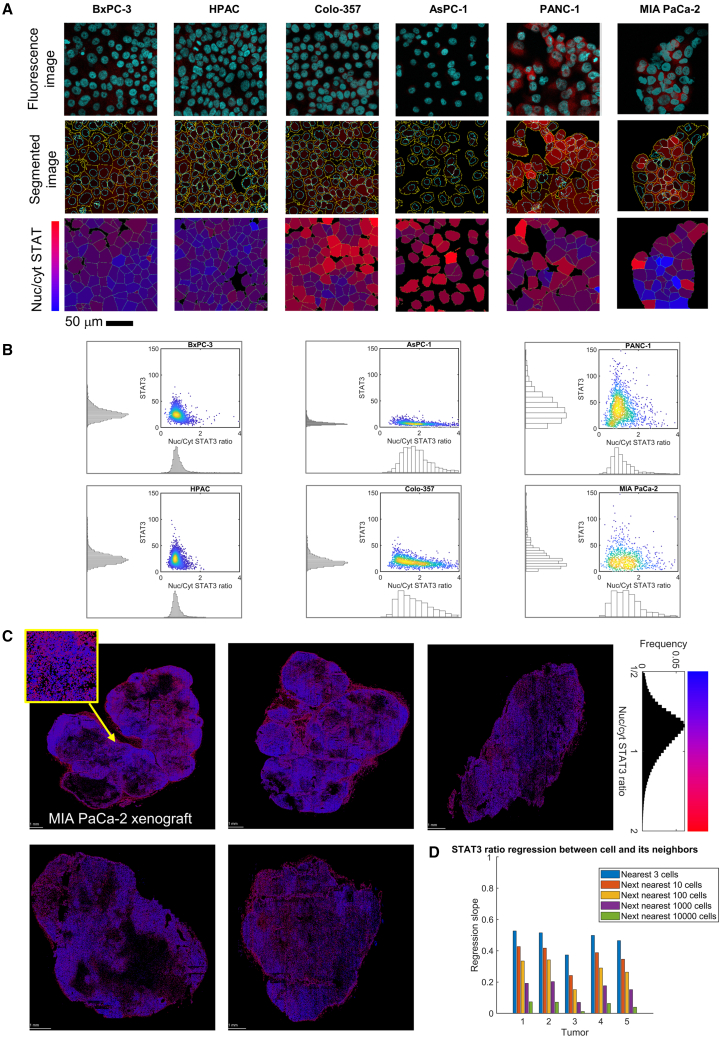
Heterogeneity of STAT3 distribution (A) Top: immunofluorescence images of PDAC monolayers showing STAT3 (red) and Hoechst (cyan). Middle: segmented images, based on cell outline and nuclear stain, identify cytoplasmic and nuclear regions. Bottom: nuclear-to-cytoplasmic ratio of STAT3 signal, analyzed on cell-by-cell basis and pseudo-colored according to shown lookup table (LUT). (B) Analysis of immunofluorescence images from at least 3 independent experiments. Imaging settings were consistent to enable between-line comparisons. MIA PaCa-2 cells show the greatest variation in STAT3 levels and its nuclear/cytoplasmic distribution. (C) MIA PaCa-2 xenografts stained for Hoechst and STAT3. Segmentation analysis measured STAT3 signal in the nucleus and its surrounding cytoplasm for all cells in the tumor section (exemplar section per tumor). Color according to nuclear/cytoplasmic STAT3 ratio. (D) Nearest-neighbor analysis, correlating STAT3 nuclear/cytoplasmic ratio to the ratio in the nearest 3 cells, next-nearest 10 cells, next-nearest 100 cells, and next-nearest 10,000 cells (Pearson’s correlation coefficient).

If STAT3 activity were a driver of metabolic heterogeneity, its immunofluorescence pattern is expected to reflect this. Six PDAC cell lines were imaged and segmented to quantify STAT3 fluorescence in cytoplasmic and nuclear regions at constant acquisition settings (Figures 5A and 5B). BxPC-3 and HPAC cells had low STAT3 levels that were distributed uniformly between the nucleus and cytoplasm. STAT3 levels were even lower in AsPC-1 and Colo-357 cells, with some degree of non-uniformity. The highest STAT3 signals were reported in PANC-1 and MIA PaCa-2 cells, of which the latter also showed evidence for a broader distribution of the nuclear/cytoplasmic STAT3 ratio. These data are consistent with measurements of fermentative rate.

Next, we investigated the state of STAT3 signaling in MIA PaCa-2 xenografts. Unlike in culture medium, where IL-6 is likely to mix uniformly, the state of IL-6R/STAT3 signaling in tumors *in vivo* may be affected by compartmentalized paracrine IL-6 signaling in a more restricted extracellular matrix. Mice were injected with MIA PaCa-2 cells subcutaneously, and tumors were grown until the humane endpoint was reached but no longer than 42 days (Figure S7C). Sections were stained using STAT3 antibody and DAPI, and cell segmentation identified cells and their nuclei. In Figure 5C, each dot represents a cell colored according to its nuclear/cytoplasmic STAT3 ratio. To quantify clustering, the STAT3 ratio for every cell was correlated with the ratio in the nearest 3 neighboring cells, and this was repeated for the next 10, 100, 1,000, and 10,000 nearest neighbors. A modest degree of clustering was observed among the 3 nearest neighbors, which dissipated over larger distances, suggesting some degree of clustering in STAT3 activation (Figure 5D).

### IL-6R/STAT3 activation is controlled by SOCS3-operated delayed negative feedback

The relationship between IL-6R and fermentation is robust because cells sorted by a proxy of elevated fermentation are enriched in *IL6R* mRNA and, conversely, because cells sorted by high surface expression of IL-6R produce a high fermentative rate. However, the state of IL-6R/STAT3 is not hardwired because sorted sub-populations return to the parental heterogeneity within a week of culture, irrespective of whether sorting was by IL-6R status or P_HLac_. This finding suggests a dynamic equilibrium between P_HLac_^high^/IL-6R^high^ and P_HLac_^low^/IL-6R^low^ cells that could be maintained by an extrinsic or intrinsic mechanism. The former could involve a secreted factor that triggers opposite responses in the two sub-populations; for example, one sub-population secretes a factor that inhibits the source cells but activates the other sub-population. If these sub-populations had contrasting metabolic rates, the system would show alternating metabolic phenotype. However, this mechanism requires the two types of cell to have hardwired, distinct transduction mechanisms, for which there is no compelling evidence in MIA PaCa-2 cells.

**Figure 6:**
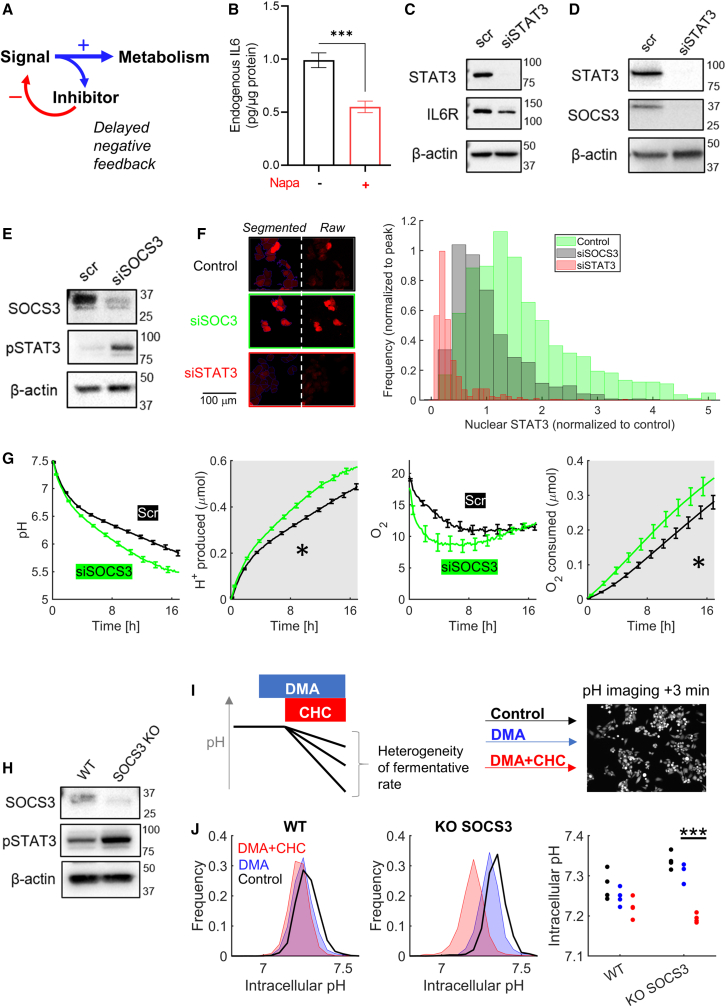
Negative feedback via SOCS3 on the IL-6R/STAT3 cascade (A) Schematic of a delayed negative feedback circuit. (B) Napabucasin (Napa) (0.2 μM, 4 h) decreases IL-6 production; assay performed on lysates (N = 4). Mean ± SEM. (C) *STAT3* knockdown reduces IL-6R expression. (D) *STAT3* knockdown reduces SOCS3 expression. (E) Effect of SOCS3 knockdown on STAT3 and pSTAT3 levels, showing negative feedback. (F) Analysis of STAT3 immunofluorescence showing effect of STAT3 or SOCS3 knockdown on nuclear STAT3 levels, alongside exemplar images (N = 4 repeats). (G) Effect of SOCS3 knockdown on fermentative and respiratory rates measured by fluorimetric assay, showing significant acceleration (N = 4; two-way ANOVA). Mean ± SEM. (H) SOCS3 knockout clone raises pSTAT3 levels. (I) Protocol for interrogating heterogeneity of fermentative metabolism in wild-type and SOCS3 knockout cells. Cells were treated with DMA (30 μM) or DMA+CHC (2 mM) for 3 min prior to imaging (cSNARF1) to obtain the pHi distribution. (J) Effect of CHC and DMA on pHi distributions, showing major acid shift upon CHC treatment in SOCS3 KO cells. Four repeats (N = ∼3,000 cells per category). Paired t test (p = 0.0072).

The alternative mechanism would implicate an intrinsic rhythm generator that controls metabolic rate. The cell cycle, an obvious candidate for rhythm generator, was discounted earlier. A minimum requirement for a simple cycling process is to feature delayed negative feedback (Figure 6A).^20^ A possible negative feedback could involve inhibition of IL-6 production by IL-6R-activated STAT3. This model was excluded because STAT3 inhibition with napabucasin decreased IL-6 production, i.e. arguing for positive feedback (Figure 6B). Another negative feedback could involve STAT3 activation causing a decrease in *IL6R* expression, but this was excluded because STAT3 knockdown decreased IL-6R levels (Figure 6C). Thus, instead of causing inhibition, STAT3 activation potentiates IL-6 signaling by increasing receptor and ligand levels, which is consistent with previous findings (IL-6R,^35^^,^^36^ IL-6^37^^,^^38^).

An alternative feedback loop could involve SOCS-family proteins that are downstream of STAT3. Studies in other cell types have shown that activated STAT3 induces *SOCS3* expression, the protein product of which inhibits Janus kinases (JAKs) responsible for phosphorylating STAT3.^39^ We confirmed this interplay in MIA PaCa-2 cells by showing that *STAT3* knockdown decreases SOCS3 levels (Figure 6D), whereas *SOCS3* knockdown increased STAT3 phosphorylation (Figures 6E and S7D) and the abundance of STAT3-positive nuclei imaged by immunofluorescence (Figure 6F). Functionally, *SOCS3* knockdown accelerated fermentative and respiratory rates, consistent with metabolic disinhibition (Figure 6G). We next tested how inactivation of this feedback affects the heterogeneity of fermentative readouts. For this, we established *SOCS3* knockout cells and confirmed that these have higher STAT phosphorylation (Figure 6H). We inferred fermentative rate from the effect of MCT inhibition with CHC on the distribution of pHi measured in monolayers. Timing was closely adhered to in order to compare pHi following the protocol shown in Figure 6I. In wild-type cells, CHC produced a modest left shift in the pHi distribution, indicating that only a minority of cells are highly metabolically active. In SOCS3 knockout cells, however, the shift was very profound, indicating that the majority of cells responded strongly to MCT inhibition (Figure 6J).

For the delayed negative feedback circuit to operate, the cascade must be triggered by IL-6, which is produced by MIA PaCa-2 cells in a STAT3-activated manner (Figure 6B). Endogenous IL-6 secretion is likely to saturate the minority of *IL6R*-expressing cells because supplementation with IL-6 or its synthetic fusion protein, hyper-IL-6 (hyIL-6), 24 prior to metabolic phenotyping had no added effect (Figure S7E).

Our results are consistent with SOCS3 overseeing an inhibitory effect on fermentative rate via pSTAT3. Since SOCS3 transcription and translation incur a time delay, the IL-6R/STAT3/SOCS3 cascade represents a delayed negative feedback circuit. Inherently, such a circuit would cause the metabolism-activating effect of IL-6 to be rhythmic, which—in turn—would produce dynamic metabolic heterogeneity observed at the population level. This would also explain why sorting MIA PaCa-2 cells by a proxy of fermentative rate metabolism produced only short-lived contrast in metabolic activity between sub-populations separated at any particular point in time.

## Discussion

This study investigated the metabolic heterogeneity in PDAC cell lines. We focused on fermentative rate because pancreatic cancers generate substantial lactic acid fluxes, linked to mutations that promote hyper-proliferation.^16^^,^^17^^,^^18^^,^^19^ A single-cell assay of fermentative rate quantified metabolic heterogeneity in a panel of PDAC lines, identifying MIA PaCa-2 as having the widest distribution. To investigate mechanisms, we tested strategies to separate cells by fermentative rate. Sorting by intracellular glucose levels, inferred using a fluorescent derivative, did not produce sufficient metabolic contrast between sub-populations. Similarly, intracellular [lactate] measured with a genetically encoded sensor was associated with inadequate power to resolve sub-populations. There are two issues with using steady-state metabolite concentrations to separate cells by metabolic phenotype: a point measurement cannot infer flux, and metabolite levels can be labile during sample preparation and sorting. We designed a novel strategy for separating cancer cells using a proxy of fermentation rate. Since glycolytic lactic acid production must be in balance with its transmembrane excretion, we reasoned that measurements of P_HLac_ are a surrogate of fermentative flux. Critically, membrane permeability properties are less labile than metabolite concentrations and relate more directly to actual flux. In support of our approach, within-population variation in P_HLac_ among PDAC cell lines followed the same rank order as variation in fermentative rate.

The validity of using P_HLac_ for sorting cells was confirmed by the metabolic contrast in the emergent sub-populations. Intriguingly, the sub-population with a high fermentative rate also had a high respiratory rate, indicating a general activation of energy-harnessing metabolism rather than a switchover. When extrapolated to the point of sorting, the difference in fermentative and respiratory rates between sub-populations was an order of magnitude. This metabolic contrast decayed with a time constant of ∼30 h, which argues against a hardwired metabolic state of P_HLac_-sorted sub-populations. Instead, the symmetrical return of the high and low sub-populations toward a mid-point metabolic rate suggests a dynamic equilibrium between cells differing in metabolic state.

Analysis of transcriptomic data from P_HLac_-sorted cells indicated no significant enrichment in stemness markers in either sub-population. Similarly, there was no enrichment in cell-cycle markers, indicating that the metabolic contrast was not because of capturing cells at different stages of their cycle. Similar metabolic contrast between sub-populations was obtained after synchronizing the cell cycle. Moreover, P_HLac_ was no different in cells in different stages of the cycle, as gated by nuclear Hoechst fluorescence or geminin signal. Despite the absence of a cell-cycle signature, the metabolically activated sub-population had faster growth post-sorting, suggesting that a conducive metabolic state drives cell division rather than vice versa (cell-cycle progression driving metabolic responses^8^).

Strikingly, cells sorted by metabolic proxies were not enriched in genes of canonical glycolytic and respiratory pathways, which highlights the limitations of making inferences on metabolic activity from gene expression. This also indicated that at least a component of metabolic control is exercised post-translationally or thermodynamically. Indeed, we found several lines of evidence associating metabolically active (P_HLac_^high^) cells with activated IL-6R/STAT3 signaling. Transcriptomics identified *IL6R* as the most significantly overexpressed gene in metabolically active cells, and pathway analysis of DEGs indicated that STAT3 was at the center of gene responses activated in P_HLac_^high^ cells. Consistent with the transient nature of the metabolic contrast, genes that were differentially expressed in sorted sub-populations progressed toward equal expression. To validate the association between IL-6R and metabolism, we found that cells sorted by high IL-6R expression also had a higher fermentative rate. In agreement with measurements of metabolic heterogeneity, MIA PaCa-2 cells showed the most substantial variation in the nuclear/cytoplasmic STAT3 ratio, an indicator of STAT3 activation. The IL-6R/STAT3/metabolism link was verified by knockdown and pharmacology. IL-6R and STAT3 knockdown as well as STAT3 inhibition (napabucasin) reduced fermentative rate; IL-6R knockdown also narrowed the P_HLac_ distribution by eliminating cells with the highest metabolic activation. Strikingly, the inhibitory effect of napabucasin on fermentative rate transiently stimulated oxygen consumption, which argues that STAT3 inhibition can selectively target fermentative metabolism rather than cause a general rundown of metabolism.

The cytokine IL-6 triggers multiple downstream responses, including metabolic regulation.^40^ Its receptor consists of the IL-6-specific receptor IL-6Rα (*IL6R*) and the widely expressed signal-transducing subunit gp130 (*IL6ST*). IL-6Rα expression is normally associated with hepatocytes and leukocytes but has also been described in cancer cells, including PDAC.^40^ Upon forming the receptor complex, JAKs phosphorylate gp130, triggering STAT3 phosphorylation to its active form. In addition to its metabolic effects, pSTAT3 induces the transcription of multiple genes, including *IL6* and *IL6R*, which has the effect of amplifying IL-6/STAT3 signaling.^41^ Another target is *SOCS3*, which inhibits the signaling between IL-6R and STAT3.^42^ Reducing SOCS3 expression increased the level of STAT3 phosphorylation and the abundance of cells with nuclear STAT3. This disinhibition also increased the bulk fermentative rate by shifting the distribution of the metabolic rate toward more active cells. Critically, SOCS3 operates a delayed negative feedback, which is one mechanism for producing rhythmic activity.^20^ The SOCS3-dependent loop incurs a delay due to transcription, translation, trafficking out of the nucleus, and inhibition of JAKs before it reduces STAT3 phosphorylation. While these parameters remain poorly defined, the scope for fine-tuning IL-6R/STAT3 rhythm may be considerable. A notable regulator is the PEST motif (a sequence rich in proline, glutamate, serine, and threonine), which facilitates SOCS3 degradation and regulates protein half-life, estimated to vary from 1^43^ to >24 h.^44^

There will be a number of reasons why some cancer cell lines manifest greater metabolic heterogeneity. In the context of IL-6R/STAT3 signaling, we propose that a necessary condition is the relatively low expression of elements of this pathway at the bulk level because this allows periods of overexpression. Indeed, MIA PaCa-2 cells differed from other PDAC cell lines in having relatively low levels of the DEGs identified in sub-populations. Cancers that have low IL-6R/STAT3 expression and delayed SOCS3 feedback may alternate metabolic activity in a manner similar to MIA PaCa-2. In contrast, lines with high population-averaged expression of the aforementioned genes may reach saturation that prevents dynamic shifts to a lower metabolic state. Functional measurements across large panels of cancer lines are required to characterize the conditions necessary for such a form of metabolic heterogeneity and to determine how widespread this phenomenon is among various types of cancer.

A role of the IL-6R/STAT3/SOCS3 cascade in driving metabolic heterogeneity is novel but does not invalidate alternative mechanisms described previously.^7^ We identified this mechanism by capturing cells at a point of elevated metabolic activity, manifested as high P_HLac_. The short-lived nature of this state may have evaded discovery in earlier studies. This cascade will have additional roles in pancreatic cancer that may benefit from delayed negative feedback. For example, IL-6 signaling facilitates the progression of pre-neoplastic lesions to malignant forms, and negative feedback by SOCS3 proteins was found to decrease primary tumor growth.^45^ The inhibitory influence of SOCS3 may vary depending on disease stage and conditions; for example, dynamic changes may become crucial in more advanced tumors, where limited resources mandate rationing.

All biological processes feature variation because “noise” is introduced across scales: from genotype to post-translational modifications. Many normal tissues impose limits on variation to stay within physiological norms; for example, excessive spread in channel activity in the heart can lead to arrhythmia.^46^ In cancers, variation is essential as a substrate for selection and disease progression. Consecutive rounds of selection in aggressive neoplasms eliminate cells with a weaker survival advantage. On this basis, low metabolic rates should be viewed as disadvantageous because they restrict proliferation; however, evidence points to a substantial level of metabolic heterogeneity even in aggressive cancers.^6^^,^^7^ Stable heterogeneity may be maintained if low-metabolic cells presented a distinct survival benefit that would keep them in balance with high-metabolic cells; however, the nature of such a benefit is elusive.^4^ Mathematical models of cancer often consider stoichiometric coupling between genotype and its phenotype, but ensemble functions, like metabolic rate, are more complex. Metabolic rate may seamlessly alternate between states, giving cancer cells a time-window for engaging energetically-demanding processes, such as protein synthesis or cell division. The state of IL-6/STAT3/SOCS signaling would manifest as dynamic, population-level heterogeneity, even among genetically identical cells. Desynchronized dynamics would ration resources evenly, while maintaining a relatively “normal” bulk metabolic rate, as shown in primary tumors.^6^ We speculate that dynamic changes in metabolic state may determine when cell division is energetically permissible because cells sorted by metabolic rate were not enriched in cell-cycle genes but proliferated faster in subsequent culture. Conversely, episodes of low activity may favor metastatic behaviors, which require time for cells to colonize new niches. Recently, Rossi et al.^47^ proposed that low phosphoglycerate dehydrogenase expression facilitates metastasis because lower glycolytic rates favor survival of circulating cancer cells. Cycles of IL-6R/STAT3 signaling may promote metastatic spread by increasing the likelihood of favorable phenotypes detaching from the primary tumor and surviving long enough to colonize distant tissues. It is noteworthy that sub-populations of cells with low fermentative rates also overexpress *GJA1*, the gene coding for Cx43 that has been linked to metastasis. Previously, we showed that Cx43 levels are low in MIA PaCa-2 primary tumors but increase in areas of invasion and metastasis.^48^ This difference may reflect a predisposition of metabolism-alternating cells to metastasize.

It will be important to demonstrate if, and how, dynamic metabolic heterogeneity supports tumor growth *in vivo*. We speculate that alternating metabolic state may benefit tumors by rationing resources among cells and therefore slowing resource depletion. Cells that are genetically instructed to have a high proliferative rate may benefit from periods of metabolic quiescence because this would mitigate against excessive resource depletion and waste buildup, i.e., risks of catastrophic cell death. Demonstrating the benefit of rationing would require paired measurements of resource consumption (input) and tissue growth (outcome) *in vivo*. If dynamic changes in metabolic rate improve the tumor’s energetic efficiency, it is reasonable to expect that they would be selected for, but this remains to be determined experimentally. Cancer-specific mechanisms that drive metabolic heterogeneity could become potential targets for therapeutic intervention; however, more research is needed.

### Limitations of the study

The scope of our investigation was limited by the low yield of cells obtained by FACS because of the short window for sorting. Engineering improvements to this workflow will be necessary to prepare sufficient material for studies such as metabolomics or proteomics that would seek differences in metabolites or post-translational modifications that fully dissect the IL-6R/STAT3 pathway. Although the link between metabolic rate, P_HLac_, and IL-6R is validated herein, there were major transcriptional differences between sub-populations of contrasting metabolic rates that may relate to pathways other than IL-6R/STAT3/SOCS3. While the IL-6R/STAT3 cascade is attractive because of its link to metabolism and its delayed negative feedback, other genes may contribute to the distinct phenotypes and warrant further testing. The mechanism described in MIA PaCa-2 cells can explain one aspect of metabolic heterogeneity in this PDAC line, but other mechanisms may be relevant in other cancer cells. Additionally, this study was limited to measurements of cells grown as 2D monolayers in an attempt to dissipate any non-uniformity of microenvironment, which could, *per se*, produce metabolic variation. In tumors, an additional layer of metabolic heterogeneity will arise because of the immediate environment of cells, such as gradients of signaling molecules.

## STAR★Methods

### Key resources table

**Table:** 

REAGENT or RESOURCE	SOURCE	IDENTIFIER
**Antibodies**		

Alexa Fluor 488-conjugated anti-rabbit	Invitrogen	A32731; RRID:AB_2633280
Alexa Fluor 555-conjugated anti-mouse	Invitrogen	A32727; RRID:AB_2633276
HRP-conjugated anti-mouse	Invitrogen	G-21040; RRID:AB_2536527
HRP-conjugated anti-rabbit	Invitrogen	G-21234; RRID:AB_2536530
HRP-conjugated anti-β-actin	Proteintech	HRP-60008; RRID:AB_2819183
IL-6 (ELISA)	Proteintech	21865-1-AP; RRID:AB_11142677
IL-6R (mask for IF)	Proteintech	23457-1-AP; RRID:AB_2827428
IL-6R (western blotting)	Santa Cruz	sc-373708; RRID:AB_10947248
PE Mouse IgG1, kappa Isotype Ctrl (FC)	Biolegend	400113; RRID:AB_326435
PE-conjugated IL6R (FACS)	Biolegend	352803; RRID:AB_10900066
Phospho-STAT3	Cell Signaling	9145S; RRID:AB_2491009
STAT3	Cell Signaling	9139S; RRID:AB_331757

**Chemicals, peptides and recombinant proteins**		

2-deoxy-2-[(7-nitro-2,1,3-benzoxadiazol-4-yl) amino]-D-glucose (2-NBDG)	Life Technologies	N13195
5-(and-6)-carboxy SNARF-1 acetoxymethyl ester, acetate	Invitrogen	C1272
5-(N,N-Dimethyl)amiloride hydrochloride (DMA)	Sigma-Aldrich	A4562
8-Hydroxypyrene-1,3,6-trisulfonic acid trisodium salt (HPTS)	Sigma-Aldrich	H1529
Acetic acid	Sigma-Aldrich	A6283
Acrylamide	Geneflow Ltd	A2-0074
alpha-cyano-4-hydroxycinnamic acid (CHC)	Selleckchem	S8612
Calcein AM	Merck Life Sciences	C1430
Cell staining buffer	Biolegend	420201
Cell tracker orange CMRA	Life Technologies	C34551
D-(+)-Galactose	Sigma-Aldrich	G5388
D-(+)-Glucose	Sigma-Aldrich	G7021
Fetal Bovine Serum (FBS)	Merck Life Science	F9665
Glutamax	Life Technologies	35050038
HEPES	Sigma-Aldrich	H3375
Hoechst 33342	Invitrogen	H3570
Human TruStain FcX	Biolegend	422302
Hydroxyurea	Sigma-Aldrich	H8627
Lipofectamine Transfection RNAiMAX reagent	Life Technologies	13778150
Marimastat	Cambridge Bioscience	14869
MES	Sigma-Aldrich	M3671
Mineral oil	Merck Life Science	M5904
MitoTracker Red	Invitrogen	M22425
napabucasin	Cambridge Bioscience	22255
Penicillin-Streptomycin	Sigma-Aldrich	P0781
Polybrene	Merck Life Science	H9268-5G
Propidium iodide	Sigma-Aldrich	P4864
Puromycin	Santa Cruz	sc-108071A
Puromycin	Santa Cruz	sc-108071A
Radioimmunoprecipitation assay (RIPA) buffer	Cell Signaling	9806S
Recombinant Human IL-6 R alpha/IL-6 Protein Chimera	Bio-Techne	8954-SR-025
Recombinant IL6	Cambridge Bioscience	009-001-310
RPMI	Merck Life Science	R0883
Sodium bicarbonate, glucose and phenol red-free DMEM	Sigma-Aldrich	D5030
Sodium bicarbonate	Sigma-Aldrich	S5761
Sodium bicarbonate-free DMEM	Sigma-Aldrich	D7777
Sodium chloride	Sigma-Aldrich	S5653
Sodium pyruvate	Gibco	11360–070
Sulphorhodamine B	Sigma-Aldrich	230162-5G
Trichloroacetic acid (TCA)	Merck Life Science	91230-100G
Tris Base	Sigma-Aldrich	T1503
Tris(bipyridine)ruthenium(II) chloride (RuBPY)	Sigma-Aldrich	224758

**Critical commercial assays**		

Bicinchoninic acid (BCA) protein assay kit	Thermo Fisher Scientific	23225
iScript cDNA Synthesis script	Bio-Rad	1708891
QIAquick Gel Extraction Kit	QIAGEN	28706X4
qRT-PCR Brilliant III SYBR Master Mix	Agilent	600886
RNeasy Micro Kit	QIAGEN	74004
TMB ELISA substrate	Abcam	ab171522
Stop Solution for TMB Substrate	Abcam	ab171529

**Deposited data**		

Results of RNAseq analysis	GEO accession viewer https://www.ncbi.nlm.nih.gov/geo/	GSE228611

**Experimental models: Cell lines**		

Human AsPC1	Prof. Anna Trauzold, University of Kiel, Germany	N/A
Human BxPC3	Prof. Anna Trauzold, University of Kiel, Germany	N/A
Human Colo-357	Prof. Anna Trauzold, University of Kiel, Germany	N/A
Human HPAC	ATCC	CRL-2119
Human: MIA Pa-Ca-2	Prof. Alessandra Fiorio, University of Lille, France	N/A
Human PANC-1	Prof. Anna Trauzold, University of Kiel, Germany	N/A

**Experimental models: Organisms/Strains**		

Female athymic Nude Crl:NU(NCr)-Foxn1nu mice	Charles River	N/A

**Oligonucleotides**		

IL-6R siRNA	Santa Cruz	sc-35663
LentiCRISPR sgSOCS3 GATGTAATAGGCTCTTCTGG	Sigma-Aldrich	N/A
LentiCRISPR sgSOCS3 TGAGCGTGAAGAAGTGGCGC	Sigma-Aldrich	N/A
siGENOME siControl	Dharmacon	D-001210-01-05
siGENOME SOCS3	Dharmacon	M-004299-02-0005
SOCS3 siRNA	Santa Cruz	SC-41000
STAT3 siRNA	Santa Cruz	sc-29493

**Bacterial and virus strains**		

5-alpha Competent E. coli (High Efficiency)	New England Biolabs	C2987H
Premo FUCCI Cell Cycle Sensor (BacMam 2.0)	Life Technologies	P36238

**Recombinant DNA**		

Laconic/pcDNA3.1(−)	San Martin et al., 2013^25^	Addgene: 44238
lentiCRISPR v2	Sanjana et al., 2014^49^	Addgene: 52961
lentiCRISPR constructs with gRNA insert listed above	This paper	N/A

**Software and algorithms**		

Fiji	ImageJ	N/A
Gen5 v.10	Biotek	N/A
MATLAB R2020b	Mathworks	N/A
Singscore R package	Foroutan et al., 2018^50^	N/A

### Resource availability

#### Lead contact

Further information and requests for resources and reagents should be directed to and will be fulfilled by the lead contact Pawel Swietach (pawel.swietach@dpag.ox.ac.uk).

#### Materials availability

This study did not generate unique reagents.

#### Data and code availability

• This paper does not contain any standardized datasets. All data reported in this paper will be shared by the lead contact upon request.
• RNAseq data have been uploaded to Gene Expression Omnibus accession number GSE228611*.*
• Any additional information required to reanalyze the data reported in this paper is available from the lead contact upon request.

### Experimental model and study participant details

#### Cell lines and culture conditions

Human Pancreatic Adenocarcinoma cell lines (listed in key resource table) were cultured in RPMI (Sigma-Aldrich, R0883) supplemented with 10% FBS, 1% penicillin–streptomycin mixture, 1% GlutaMAX (35050-038, Gibco, Waltham, MA, USA), 1% sodium pyruvate (11360-039, Gibco, Waltham, MA, USA). In the case of media incubated in a 5% CO2-enriched atmosphere, pH buffering was provided by CO2/HCO3- and the appropriate [HCO3-] was determined empirically to equilibrate at the target pH 49. Cell lines authentication based on Short Tandem Repeat (STR) profiling was conducted in the AuthentiCell service provided by the European Collection of Authenticated Cell Cultures.

#### Animals

All animal procedures followed the Animals (Scientific Procedures) Act 1986 and were authorized by Project License no. PPL P01A04016. To assess the levels of cell heterogeneity *in vivo*, five female athymic Nude Crl:NU(NCr)-Foxn1nu mice received a subcutaneous injection of MIA PaCa-2 cell suspension as 100 μL of a 1:1 mixture of Matrigel and serum-free DMEM medium. Each mouse was injected with 2 million MIA PaCa-2 cells on the left flank. The animals were sacrificed when the tumor size reached the humane endpoint, and the tumor samples were collected into PBS and processed for immunohistochemical analysis.

### Method details

#### Cell cycle synchronization

MIA PaCa-2 cells were synchronized using hydoxyurea-induced arrest in G1/S phase according to a published protocol.^51^ Briefly, the cells were plated onto 10 cm dishes and left to attach overnight. Next day, the standard culture medium was replaced with FBS-free and maintained for 24 h. Then, the medium was replaced with fresh FBS-containing medium with the addition of 4 mM hydroxyurea for 24 h. After incubation the cells were collected for cell cycle analysis and FACS followed by metabolic profiling.

#### Cell cycle analysis

HU-synchronised and control cells were collected, washed with PBS and fixed by incubating with ice-cold 70% ethanol for 30 min at 4°C. Next, the cells were washed twice with PBS, treated with RNAse and stained with the propidium iodide. The samples were analyzed using BD LSRFortessa (Dunn School of Pathology), and cell aggregates were excluded from the analysis.

#### Flow cytometry cell sorting based on glucose uptake capacity

To assess the relationship between glucose uptake capacity and glycolytic rate, MIA PaCa-2 cells were collected and washed twice in cell staining buffer. Then, the cells were centrifuged, and the pellet was resuspended in cell staining buffer (BioLegend, #420201) containing 50 μM fluorescent glucose analog (2-NBDG, Life Technologies, #N13195) and incubated for 15 min at room temperature. Then, the cells were centrifuged, resuspended in cell staining buffer, and filtered prior to sorting. The samples were sorted using BD FACSAria III Cell Sorter (Dunn School of Pathology), the fluorescence of the 2-NBDG was excited at 488 and the emission was measured at 530 nm. The dead, DAPI-stained cells, and cell doublets were excluded from the gating. The collected cells were plated in equal number for metabolic phenotype assessment at 24 h after sorting.

#### Fluorimetric assay of glycolytic and respiratory rate

This dual-dye fluorimetric assay was based on a recently published method.^24^ Cells were seeded in a 96-well plate at 70k/per well (or lower, but equal density between the conditions when sorted cells were seeded). The medium was buffered with 2 mM HEPES/2 mM MES. pH was measured using HTPS (2 μM) and oxygen tension was inferred from RuBPY (50 μM). Prior to measurements, media were covered with a layer of 150 μL mineral oil *(M5904, Sigma)* to impose a barrier to gas diffusion. Cumulative H^+^ production and O_2_ consumption were measured according to equations described previously.^24^ The plate was placed immediately in a Biotek Cytation 5 plate reader pre-heated to 37°C, recording HPTS and RuBPY fluorescence in sequential mode.

#### Superfusion

Cells were seeded at 70k per well in 4-chamber Nunc Lab-Tek slides (734–2060, ThermoScientific). Experiments involving superfusion were performed on a Leica LCS confocal microscope. Intracellular pH was measured using cSNARF1 (17 μM for 10 min; C1272 ThermoFisher), excited at 514 nm and collected at 580 nm and 640 nm. The ratio was calibrated to units of pH using a calibration curve determined in separate experiments using the nigericin technique.^52^ Solutions were delivered by a system of tubes operated by a peristaltic pump, with a two-level solution switcher to alternate between one of two superfusates heated to 37°C. Excess solution was drawn by a vacuum pump to ensure laminar flow. Solutions were based on normal Tyrode containing 4.5 mM KCl, 1 mM CaCl2, 1 mM MgCl2, 11 mM glucose, 10 mM HEPES, 10 mM MES and 130 mM NaCl, titrated to a desired pH (5.7–7.7) using HCl or NaOH.

#### Rapid solution switching

cSNARF1-loaded PDAC cells seeded in Lab-Tek chambers were superfused with normal Tyrode. A dual microperfusion device was manipulated into the field of view, 100 μm from the cells of interest. Switching a system of valves alternated between the two microstreams, driven by gravity flow. The speed of solution switching is 25 ms.^53^ For the single-cell glycolytic rate assay, the first microstream contained normal Tyrode and the second was supplemented with 2 mM α-cyano-*4*-hydroxycinnamate (CHC; S8612, Selleckchem) to block lactic acid efflux by MCT. Both microstreams containing 30 μM 5-N,N-dimethylamiloride to block acid-extrusion by Na^+^/H^+^ exchanger-1. To evoke MCT activity, the first microstream contained 30 mM L-lactate and the second was lactate-free, both titrated to pH 7.4. Cells were equilibrated with the lactate microstream for 5 min to load cytoplasm with MCT substrate, and then lactate washout triggered lactic acid efflux, principally as H + -lactate via MCT. The rate of pHi change multiplied by buffering capacity informed flux, which was converted to permeability PMCT after calculating the driving force.

#### Tracking cell cycle with GFP-tagged geminin

Cells seeded in Labtek slides were treated with 28 μL of geminin-GFP reagent, one of two components of Premo FUCCI Cell Cycle Sensor BacMam 2.0 (P36238, Invitrogen) added to 1mL medium, and incubated for 16 h. The other component, Cdt1-RFP was omitted due to spectral overlap with cSNARF1. The medium was then changed for HEPES-buffered RPMI (R7388) containing cSNARF1 (17 μM; C1272 ThermoFisher) and Hoechst (3.3 μM; 34580) for 10 min cSNARF1 fluorescence (excitation 515 nm, emission 580/640 nm) identified cytoplasm. This was used to measure, sequentially, the total Hoechst signal (excitation 361 nm, emission 450 nm) and mean GFP signal (excitation 488 nm, emission 520 nm).

#### Flow cytometric cell sorting by MCT activity

Suspensions of MIA PaCa-2 cells were loaded with cSNARF1 for 10 min and equilibrated with 60 mM L-lactate in 10 mM HEPES/10 mM MES-buffered, FCS-free medium for 5 min. Cells were then spun down and re-suspended in a lactate-free formulation of the medium, and immediately subjected to sorting based on the intracellular pH on a BD FACSAria III. The sorting period was limited to 10 min, which adequately distinguishes cells with high and low MCT permeability (alkaline and acidic pHi). Fluorescence was excited at 561 nm and measured at 582 nm or 670 nm, which produces a pH-sensitive ratio.

#### Sulforhodamine B (SRB) assay for cell growth

Cells were seeded in 96-well plates at 10k/well and left to adhere for 24 h. After this, the medium was changed to the desired composition, and cells incubated for 4 days in 5% CO2. Plates were fixed by adding 100 μL 10% tricarbolic acid and incubated for 1h at 4°C, washed 4 times with water, stained with SRB (0.057% in 1% acetic acid, #230162, Sigma-Aldrich) for 30 min at RT, washed off 4 times with 1% acetic acid, left to dry, and the crystals were dissolved in 200 μL 10 mM Tris. The colorimetric measurement of dissolved SRB was done using Cytation 5 plate reader, as described previously.^54^

#### Flow cytometric cell sorting by IL6R expression

For extracellular IL6R staining suspensions of MIA PaCa-2 cells were centrifuged at 300 g for 5 min and washed twice in an ice-cold Staining Solution. The non-specific binding was blocked by incubation with Human TruStain FcX (BioLegend, #163403). Next, the cells were stained with PE-conjugated anti-human CD126 antibody (BioLegend, #352803), or PE-conjugated IgG1 isotype control (BioLegend, #400113) suspended in 100 μL of 3% FBS in PBS at 4°C, in the dark for 25 min. After staining, the cells were washed twice and resuspended in staining solution, and sorted immediately using BD FACSAria III Cell Sorter. The dead, DAPI-stained cells, and cell doublets were excluded from the gating. The cells were sorted based on the IL6R fluorescence into positive and negative populations and maintained in culture for metabolic phenotype assessment after 24 h, 1, and 2 weeks after sorting.

#### RNA sequencing

The RNA from sorted cells was extracted using column-based RNeasy Micro Kit (Qiagen) according to the manufacturer’s protocol. Once eluted, the RNA samples were stored at −80°C for future experiments. The 500 μg of each sample was submitted to Oxford Genomic Center for library preparation and RNA sequencing. Briefly, the mRNA fraction was selected from the total RNA and then converted to cDNA. Second strand cDNA synthesis incorporated dUTP. The cDNA was end-repaired, A-tailed and adapter-ligated. Prior to amplification, samples were digested with uridine. The prepared libraries were size selected, multiplexed and their quality was checked before paired-end sequencing. Data were aligned to the reference human genome. The obtained data were analyzed in MATLAB using the DESeq2 package. RNAseq data have been uploaded to Gene Expression Omnibus accession number GSE228611. The enrichment of stem cell markers in PDAC was calculated using the Singscore R package53. The normalized mRNA counts of individual samples were ranked and scored for enrichment of the PDAC stemness markers^55^: NES, MYC, KLF4, CD24, CXCR4, CD44, ABCG2, OCT4, SOX2, PROM1, ALDH1A1, EPCAM, and MET. The significance was determined with Wilcoxon signed-ranks test.

#### RT-qPCR

cDNA was prepared using iScript cDNA Synthesis Kit (Bio-Rad) according to the manufacturer’s protocol. Quantitative reverse transcription PCR was conducted in technical triplicates using specific primer pairs and the Brilliant III Ultra-Fast SYBR Green qPCR Master Mix (Agilent) on an AriaMX Real-Time qPCR Instrument (Department of Oncology). Results were expressed as mean fold change compared with control using the ΔΔCt method, from four biological repeats. The expression of glyceraldehyde-3-phosphate dehydrogenase (GAPDH) and SNW Domain Containing 1 (SNW1) were used for internal calibration.

**Table:** 

	Forward	Reverse
*EFNB2*	ATGCAGAACTGCGATTTCCA	GTCCTTGTCCAGGTAGAAATTTGG
*IL6R*	CCCCACTCCTGGAACTCATC	GGAGGTCCTTGACCATCCAT
*FOXJ1*	GTGGGAGCAACTTCTTCCAGA	ATAAGTATGTGGTGCCTGGCT
*NFATC2*	AGACGAGCTTGACTTCTCCA	TGCATTCGGCTCTTCTTCGT
*USP38*	CCAGAGGCGTTCCATTTGATTG	GCTGTACTTGAAGGCAGACCA
*BAG3*	AAACAGTGTGGACAGGTGGC	GGAGACTGGGACCGCTCA
*PDP1*	TGGAAAGAGCGCCGAGC	CTTCTGACTGGGATTCCGGG
*HBEGF*	AGGAGAGGAGGTTATGATGTGGA	CCAGCCGATTCCTTGAGCA
*IL6ST*	GTGAGTGGGATGGTGGAAGG	ACTTGTGTGTTGCCCATTCAG
*SLC16A1*	CCACCACTTTTAGGTCGGCT	ATTAGGACGACGCCACATGC
*GAPDH*	TCGGAGTCAACGGATTTGGT	TGAAGGGGTCATTGATGGCA
*SNW1*	GTATCACAGAAGGTCGCCGC	GCCACTCCTTGCTGAGATGG

#### Immunoblotting

The immunoblotting was conducted as previously described. Briefly, samples were lysed in radioimmunoprecipitation assay (RIPA) buffer. Protein concentration was measured using bicinchoninic acid (BCA) protein assay kit according to the manufacturer’s protocol. Samples of equal protein content were loaded onto a 12% acrylamide gel and run at 100 V for 60 min until resolved. Afterward, proteins were transferred onto the PVDF membrane, and the non-specific binding was blocked by incubation with 5% milk for 1h at RT. The primary antibodies were raised against IL6R (Santa-Cruz, #sc-373708, 1:1000), STAT3 (Cell Signaling, #124H6, 1:1000) and pSTAT3 (Cell Signaling, #9145, 1:2000). β-actin (1:5000, Proteintech, #HRP-60008) protein was used as a loading control.

#### siRNA transfection

Cells were seeded at a density of 150,000 cells/well in a 6-well plate and transfected with targeted siRNA against IL6R (Santa-Cruz, #sc-35663, 10 nM), STAT3 (Santa-Cruz, #sc-29493, 10 nM), SOCS1 (Dharmacon, #M-011511-04-0005, 10 nM), SOCS3 (Dharmacon, # M-004299-02-0005, 10 nM), a combination of SOCS1 and SOCS3 or with a scramble non-targeted siRNA (Dharmacon, #D-001210-01-05, 10 nM), using Lipofectamine RNAiMAX (Invitrogen, #2373383). After 72 h, cells were harvested and lysed or seeded for further experiments.

#### CRISPR/Cas9-mediated SOCS3 knock-out

Expression of SOCS3 in MIA PaCa-2 cells was knocked-out as previously described.^56^ LentiCRISPR v2 plasmid was a gift from Feng Zhang (Addgene plasmid # 52961; http://n2t.net/addgene:52961; RRID:Addgene_52961). sgRNA against SOCS3 were cloned into LentiCRISPR v.2 backbone using the following protocol^49^*:* http://genome-engineering.org/gecko/wp-content/uploads/2013/12/lentiCRISPRv2-and-lenti Guide-oligo-cloning-protocol.pdf.

sgRNA sequences were as follows: GATGTAATAGGCTCTTCTGG; TGAGCGTGAAGAAGTGGCGC. Virus aliquots were prepared, and MIA PaCa-2 cells were seeded onto a 6-well plate at a density of 150,000 cells/well and transduced with a 500 mL aliquot of lentivirus carrying the LentiCRISPR v2 construct encoding an sgRNA sequence targeting SOCS3 with addition of polybrene at 4 mg/mL. The cells were maintained for two days before puromycin selection, and then seeded for clonal selection. SOCS3 knock-out was confirmed by Western blotting.

#### Enzyme-linked immunosorbent assay

The endogenous IL6 synthesis was measured in MIA PaCa-2 and PANC-1 cell lysates. Equal amounts of protein were added to high absorption 96-well plate and air-dried at 37°C overnight. Next, the plate was washed with 0.05% phosphate-buffered saline (PBS) with Tween (PBST), blocked with 3% bovine serum albumin (BSA) in PBS for 2 h, and incubated with primary anti-IL-6 antibody (Proteintech, #21865-1-AP, 1:400) for 1 h at room temperature. Then, the plate was washed four times with PBST, and incubated with HRP-conjugated anti-rabbit secondary antibody (Invitrogen, #G21234, 1:7000) for 1 h at room temperature. For the detection of IL6, 100 μL of TMB substrate was added per well (Abcam, #ab171522). After 90 s the reaction was stopped by adding 100 μL stop solution per well (Abcam, #ab171529), and the color intensity was measured at 590 nm on a Cytation 5 plate reader.

#### Immunofluorescence

The heterogeneity of STAT3 expression in PDAC cell lines was assessed via immunofluorescent staining. 100,000 cells were seeded onto 12-well Ibidi chamber slides and left to adhere overnight. Next day, the cells were fixed with 4% cold paraformaldehyde solution and incubated for 15 min at RT. The fixative was discarded, and the wells were washed three times with PBS. The non-specific binding was blocked by 1 h incubation with 3% bovine serum albumin (BSA) in PBS at room temperature. Then, the cells were incubated with a mixture of primary antibodies against IL6R (used as a cytoplasmic mask) and STAT3 dissolved in 3% BSA for 1 h at room temperature (IL6R: 1:200, Proteintech, #23457-1-AP; STAT-3: 1:1000, Cell Signaling, #124H6). Then, the slides were washed and incubated with fluorophore-conjugated secondary antibodies at 1:1000 dilution for 1 h at RT (Alexa Fluor 488, Invitrogen, #A32731; Alexa Fluor 555, Invitrogen, #A32727) Next, the nuclei were stained with Hoechst for 10 min, the slide was sealed with a mounting medium and left to dry prior to imaging.

#### Immunohistochemistry

Upon excision, tumors were washed twice in PBS and formalin-fixed at 4°C for 24 h. Next, the tumors were embedded in paraffin and cut into 4 μm sections. Prior to staining the sections were deparaffinized in xylene and rehydrated by immersion in the range of ethanol solutions of decreasing alcohol concentrations (100%, 100%, 95%, 70%, 50%) and then briefly washed in deionized water. Next, the sections were boiled in sodium citrate solution titrated to pH 6 for 20 min and rinsed in deionized water for 5 min. Then, the cells were permeabilized with 0.2% Triton X- in PBST and washed in dH2O. The tissue was circled with a hydrophobic pen and the sections were washed twice with PBST. The non-specific binding was blocked by incubation with a mixture of 2% bovine serum albumin and 0.3 M glycine dissolved in PBST for 1 h at RT. After blocking, the tissue was incubated with primary antibody against STAT3 (Cell Signaling, #124H6, 1:500) dissolved in 1% BSA/PBST for 1 h at RT. After that, the samples were washed three times in PBST and incubated with fluorophore-conjugated secondary antibody (1:1000) for 1h at room temperature (Alexa Fluor 555 anti-mouse, Invitrogen, #A32727). Finally, the sections were washed in PBST, and then in PBS and the nuclei were stained with Hoechst 33342 (1:1000). The samples were rinsed in deionized water, dried, and mounted with coverslips using Prolong Diamond antifade mounting solution. For analysis, images were taken at settings corresponding to DAPI and Alexa 555. The former was used to define the nuclear regions and the latter for cell outline. Together, these data were used to segment cells and define their nuclear and cytoplasmic regions. For each cell, STAT3 mean fluorescence in cytoplasm and nucleus was measured and ratioed, and then presented as a pseudo-colored image, where each point represents a cell and is colored by its nuclear/cytoplasmic ratio. Nearest neighbors were identified by proximity and correlation was calculated using Spearman’s coefficient for each cell in the field of view.

### Quantification and statistical analysis

#### Statistical analysis

Data were analyzed using MATLAB R2020b and GraphPad Prism 9. Results are shown as mean ± S.E.M. unless indicated otherwise. The number of observations is described as “n/N”, indicating the number of cells or wells and the number of biological repeats. The data were compared using unpaired t test, one-way or two-way ANOVA. Additional tests used to determine significance are described in figure legends. ∗ = p < 0.05, ∗∗ = p < 0.01, ∗∗∗ = p < 0.001.

## Supplemental information

Document S1. Table S1 and Figures S1–S7

Table S2. List of differentially expressed genes detected in P_HLac_^low^ and P_HLac_^high^ populations

Document S2. Article plus supplemental information
