# Supplementary material for: Dynamic IL-6R/STAT3 signaling leads to heterogeneity of metabolic phenotype in pancreatic ductal adenocarcinoma cells
Source: Cell Rep. 2023 Dec 23;43(1):113612. doi: 10.1016/j.celrep.2023.113612 (PMC11149489; doi:10.1016/j.celrep.2023.113612)

**Cell Reports, Volume *43***

## Supplemental information

# Dynamic IL-6R/STAT3 signaling leads to heterogeneity of metabolic phenotype in pancreatic ductal adenocarcinoma cells

**Wiktoria Blaszczak, Bobby White, Stefania Monterisi, and Pawel Swietach**

**Table S1. Results of short-tandem repeats (STR) profiling of PANC-1, MIA PaCa-2 and BxPC3 cells, related to Figure 1.**

| STR Locus | Test Sample: PANC-1 | Cellosaurus PANC-1: CVCL. 0480 | STR Locus | Test Sample*:* MIA PaCa-2 | Cellosaurus MIA PaCa-2: CVCL_0428 | STR Locus: | Test Sample BxPC-3 | Cellosaurus BxPC-3: CVCL0186 |
|---|---|---|---|---|---|---|---|---|
| AMEL | X | X | AMEL | X | X | AMEL | X | X |
| CSFIPO | 10,12 | 10,12 | CSFIPO | 10 | 10 | CSF1PO | 13 | 13 |
| D135317 | 11 | 11 | D13S317 | 12,13 | 12,13 | D135317 | 11 | 11 |
| D16S539 | 11 | 11 | D16S539 | 10,13 | 10,13 | D165539 | 9,11 | 9,11 |
| D18S51 | 12 | 12 | D18S51 | 12 | 12 | D18551 | 12 | 12 |
| D21511 | 28 | 28 | D21S11 | 29.31.2 | 29,31.2 | D21511 | 29 | 29 |
| D3S1358 | 17 | 17 | D3S1358 | 16 | 16 | D3S1358 | 14,16 | 14,16 |
| D5S818 | 11,13 | 11,13 | D58818 | 12,13 | 12,13 | D5SB18 | 11 | 11 |
| D7SB20 | 8,10 | 8,10 | D7S820 | 12,13 | 12,13 | D75820 | 10,13 | 10,13 |
| D8S1179 | 14,15 | 14,15 | D8S1179 | 16 | 16 | D8S1179 | 13 | 13 |
| FGA | 21 | 21 | FGA | 22 | 22 | FGA | 20,21 | 20,21 |
| PENTA D | 14 | 14 | PENTA D | 12,16 | 12,16 | PENTA D | 14 | 14 |
| PENTA E | 7,14 | 7,14 | PENTA E | 13,18 | 13,18 | PENTA E | 12,14 | 12,14 |
| THO1 | 7,8 | 7,8 | THO1 | 9,10 | 9,10 | THO1 | 9 | 9 |
| TPOX | 8,11 | 8,11 | TPOX | 9 | 9 | TPOX | 8 | 8 |
| VWA | 15 | 15 | VWA | 14,15 | 15 | VWA | 14,18 | 14,18 |

**Table S2. List of DEGs, related to Figure 3C** (csv attached). Obtained by DESeq2 analysis of four paired RNAseq datasets.

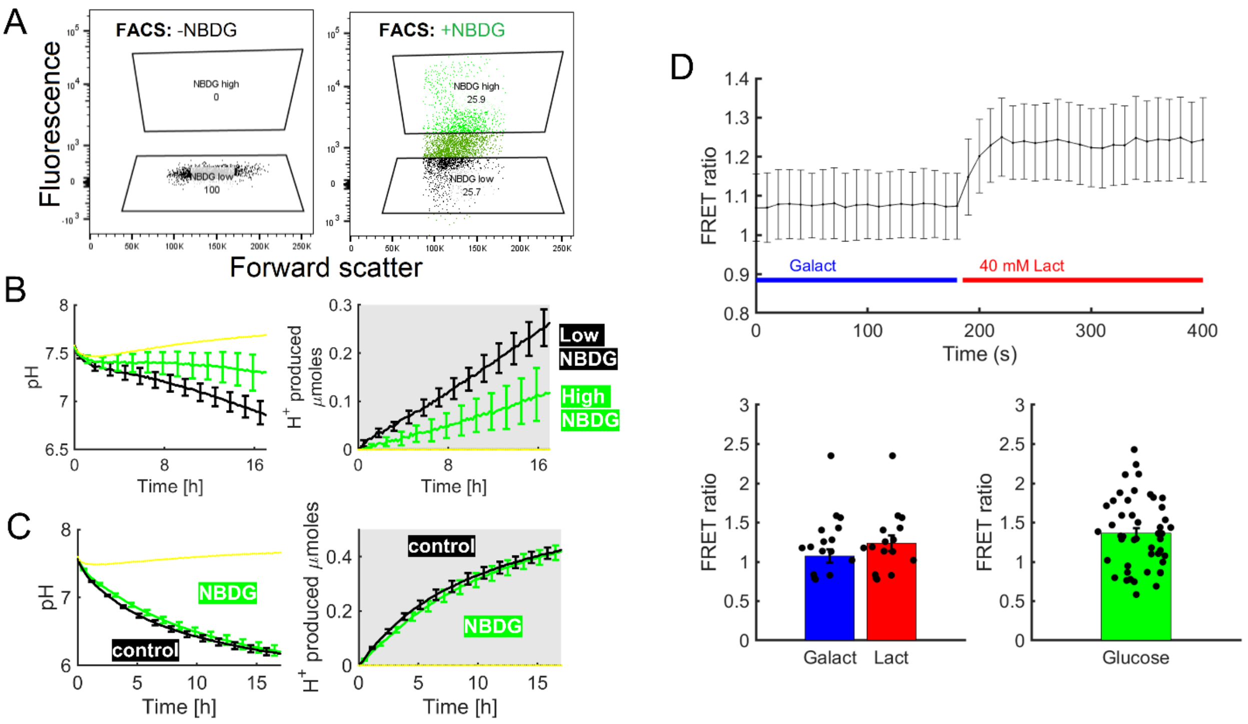


**Figure S1. Sorting cells by steady-state concentrations of metabolites or their derivatives, related to Figure 2B.** A. Sorting MIA PaCa-2 cells that had been loaded for 15 min with 50 µM 2-NBDG, a fluorescent glucose derivative. Excitation at 488 nm/emission 520 nm. B. Medium pH and fermentative rate in sub-populations obtained after sorting for 2-NBDG fluorescence. Assay performed 24 h after sorting (17k cells/well; 5 repeats). No significant difference between emergent sub-populations, indicating poor ability of 2-NBDG to separate cells by metabolic rate. C. MIA PaCa-2 cells (50k/well) assayed for fermentative rate using HPTS assay, with or without a prior period of 2-NBDG loading. Plots below show a mean of three biological repeats. 2-NBDG does not affect fermentative rate. D. MIA PaCa-2 cells transfected with Laconic. Fluorescent cells were analysed for FRET ratio. Superfusion protocol involved a switch from galactose-containing medium to lactate-containing medium. Galactose results in minimal lactate production, whereas 40 mM lactate will activate the sensor. The effect size was modest, as expected for a FRET sensor, but comparable to baseline variation. For cells superfused in glucose, the low signal-to-noise ratio is inadequate for sorting.

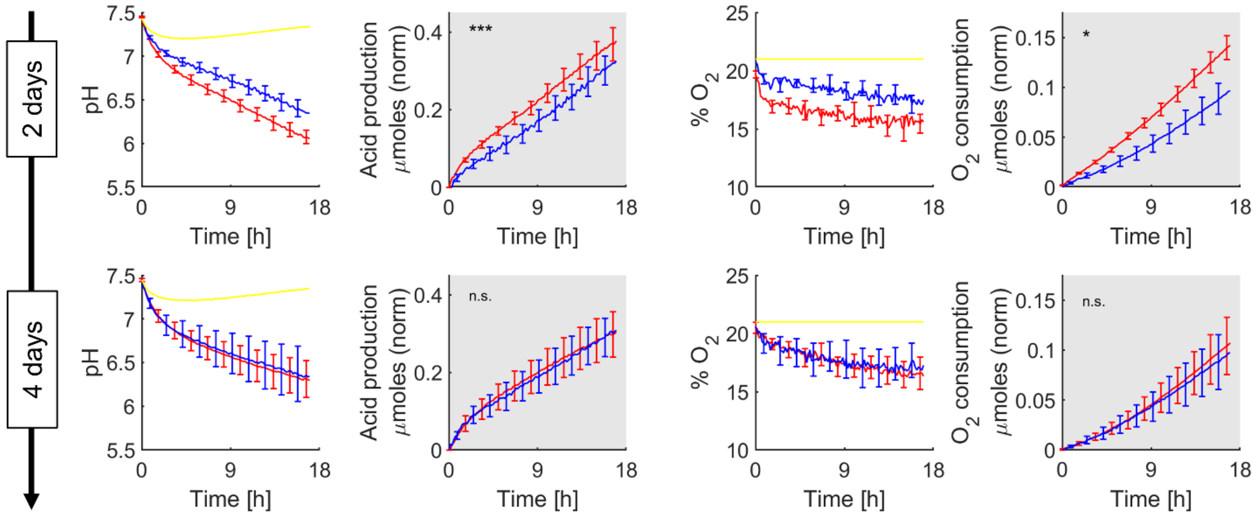


**Figure S2: Phenotyping sub-populations gated by $P_{HLac}$ related to Figure 2.** *Left:* Fluorimetric assay of fermentation rate in $P_{HLac}^{high}$ and $P_{HLac}^{low}$ sub-populations, measured after 2 or 4 days in culture post-sorting (n=12/N=4 for each). Medium pH and cumulative acid production calculated from pH time-course. Significant difference (P<0.001) between $P_{HLac}^{high}/P_{HLac}^{low}$ after 1 day of sorting (two-way ANOVA). *Right:* Medium dissolved $O_2$ and cumulative oxygen consumption calculated from $O_2$ time-course.

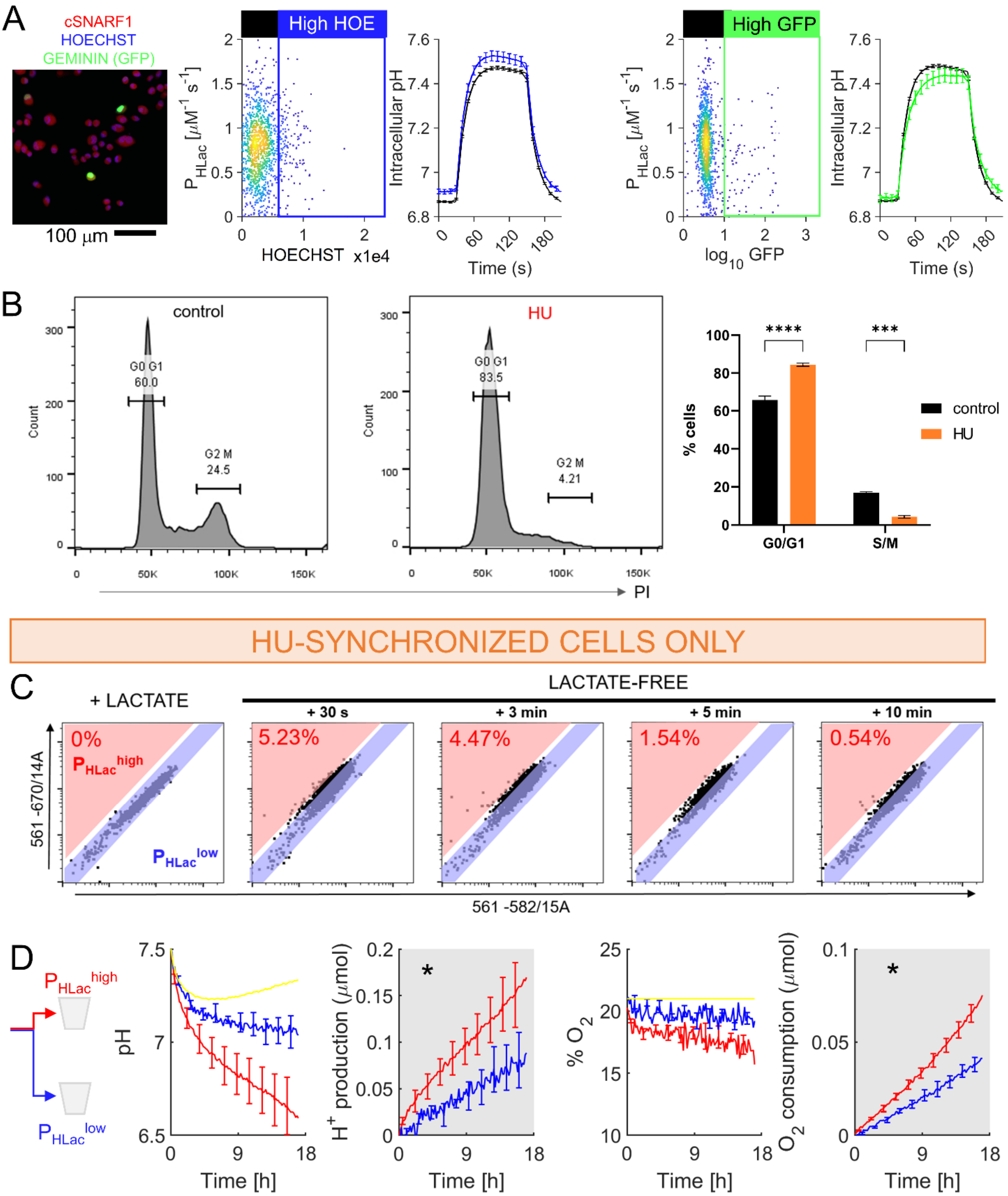


**Figure S3: Relationship between metabolic heterogeneity and the cell cycle, related to Figure 2.** A. Image of MIA PaCa-2 cells expressing GFP-tagged geminin and loaded with cSNARF1 (red) and Hoechst 33342 (blue). $P_{HLac}$ plotted as a function of total Hoechst fluorescence or mean GFP fluorescence. Mean time courses of pHi for cell sub-populations gated by Hoechst or GFP signal. Results from 3 biological repeats. B. Flow cytometric analysis of cell cycle (Hoechst) of control and hydroxyurea (HU) treated cells. C. HU treatment synchronizes cell cycle by reducing abundance in G2/M phase. D. Sorting of HU-synchronized cells produces sub-populations with contrasting fermentative and respiratory rates, similar to results obtained from control cells. Thus, the cell cycle does not affect metabolic heterogeneity, as determined by sorting cells according to $P_{HLac}$.

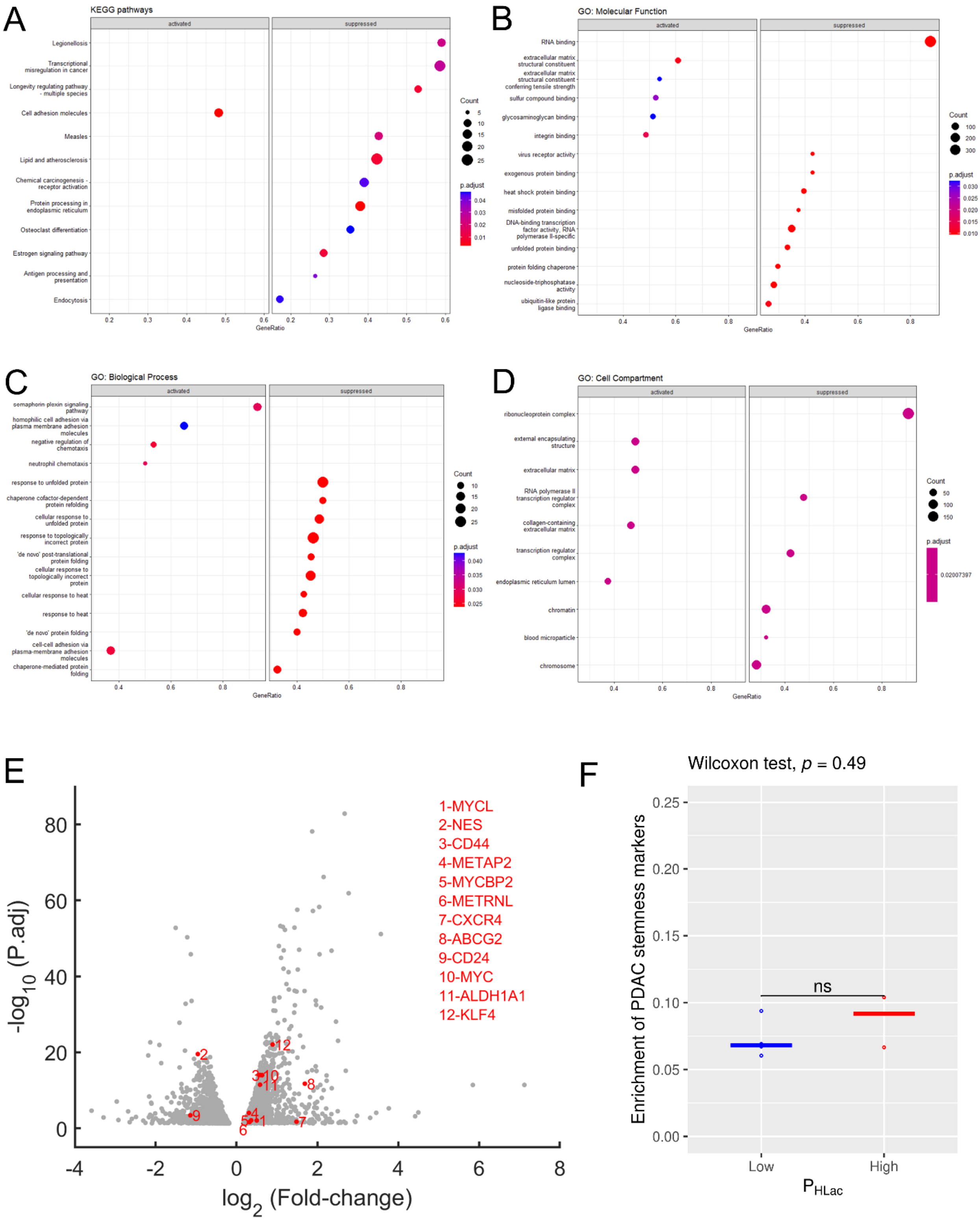


**Figure S4. Pathways, related to Figure 3.** Gene set enrichment analysis produced by clusterProfiler using DEGs identified in Figure 3. A. KEGG pathways; B-D Gene ontology. E. Analysis of stem cell markers among DEGs. F: The normalized mRNA counts of individual samples ranked and scored for enrichment of the PDAC stemness markers: NES, MYC, KLF4, CD24, CXCR4, CD44, ABCG2, OCT4, SOX2, PROM1, ALDH1A1, EPCAM, and MET. Wilcoxon signed-ranks test. p=0.486 (non-significant).

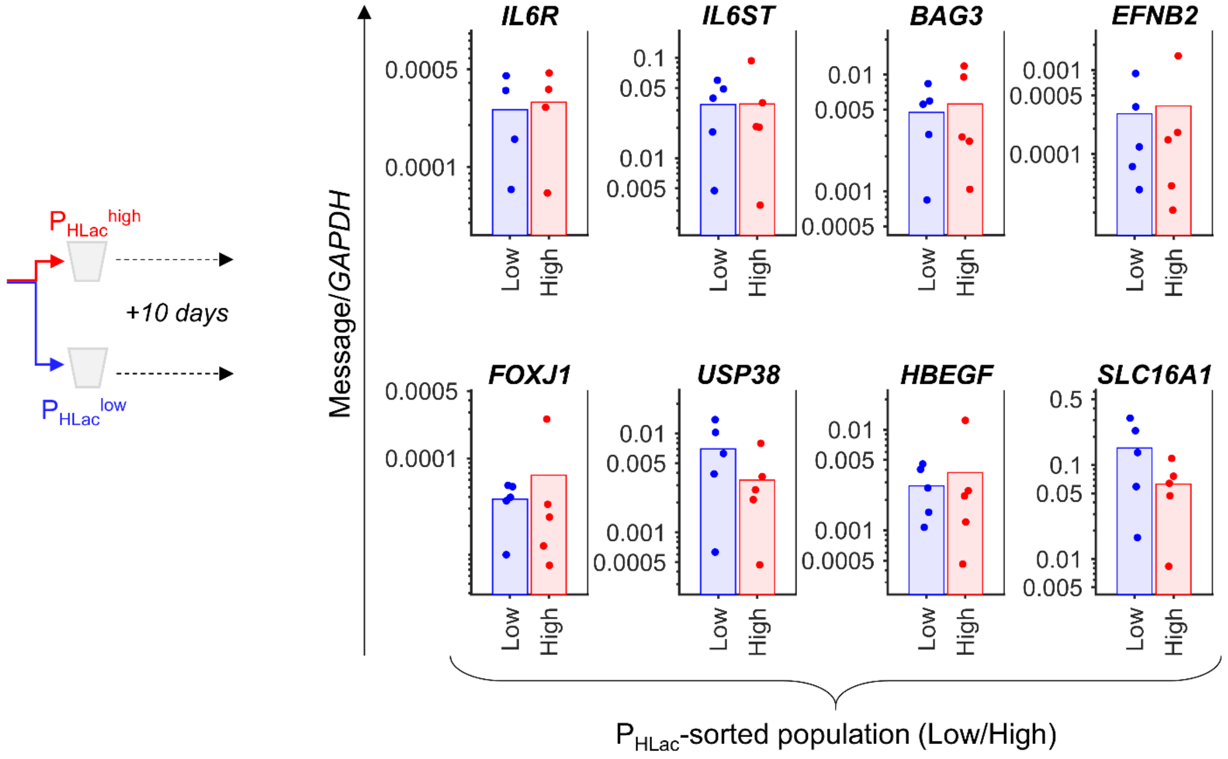


**Figure S5: Expression of genes associated with metabolic heterogeneity, related to Figure 3F.** MIA PaCa-2 cells were sorted by $P_{HLac}$ and cultured for 10 days, during which the metabolic contrast is expected to collapse. RT-qPCR confirmation of selected genes, normalized to GAPDH. No significant differences in expression (N=5).

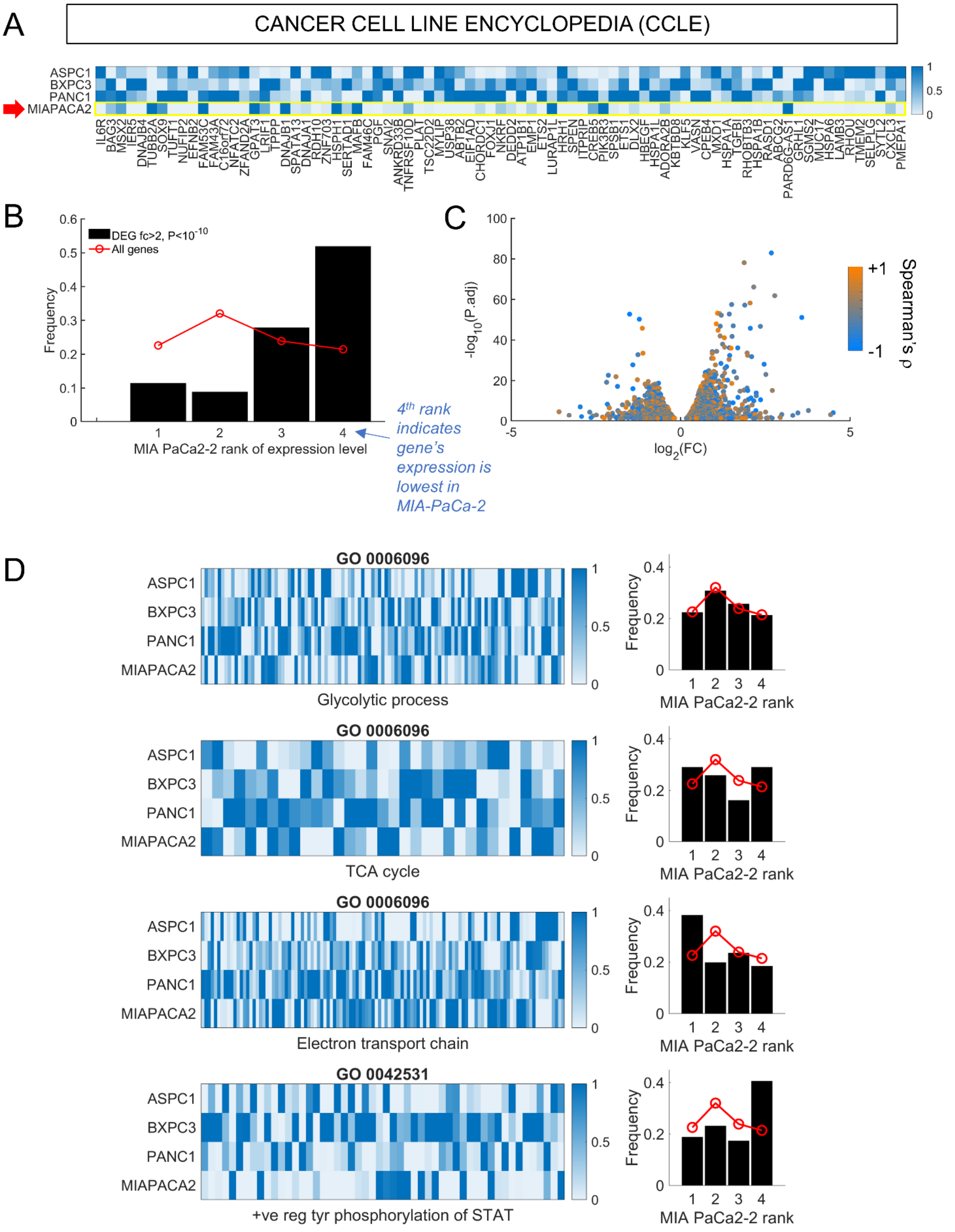


**Figure S6. CCLE analysis, related to Figure 3.** A. Analysis of CCLE RNAseq datasets for four available PDAC cell lines, ranked by increasing measure of metabolic heterogeneity: AsPC1 < BxPC3 < PANC1 < MIA PaCa2. Expression pattern of the most significantly upregulated DEGs ($P<10^{-10}$, fold-change>2) identified in $P_{HLac}^{high}$ cells. Heatmap shows normalized expression of these DEGs in "bulk" PDAC samples. Note the lowest levels of "bulk" expression in MIA PaCa-2 cells. B. The four PDAC lines were ranked by expression of genes (DEGs in

black, all genes in red). The frequency of rank is plotted on the x-axis (4th means lowest rank of the four lines). For most DEGs, expression was lowest (4th rank) in “bulk” MIA PaCa2 samples. C. Volcano plot based in Figure 3, color-coded according to the correlation between a particular gene in “bulk” RNAseq datasets and apparent metabolic heterogeneity.  Here, blue indicates genes that had the lowest “bulk” expression in MIA PaCa-2 cells. D. Heatmaps show normalized expression of genes belonging to the indicated gene ontologies.  Histograms on right show rank analysis.

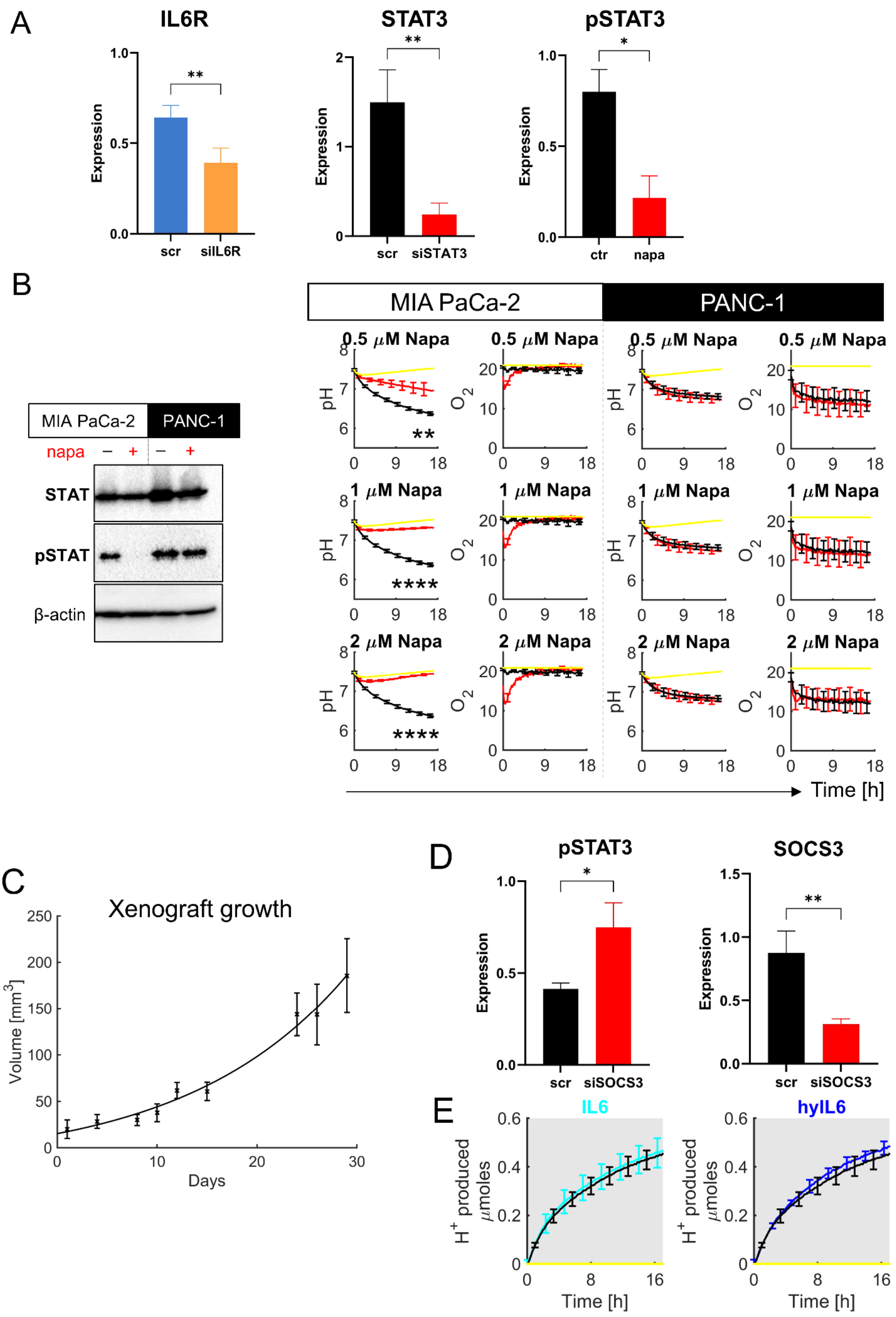
A
IL6R
Expression
1.0
0.5
0.0
**
scr
siIL6R
STAT3
Expression
2
1
0
**
scr
siSTAT3
pSTAT3
Expression
1.0
0.5
0.0
*
ctr
napa
B
MIA PaCa-2
PANC-1
napa
–
+
–
+
STAT
pSTAT
β-actin
MIA PaCa-2
PANC-1
0.5 μM Napa
1 μM Napa
2 μM Napa
pH
O2
**
****
****
Time [h]
C
Xenograft growth
Volume [mm3]
250
200
150
100
50
0
0
10
20
30
Days
D
pSTAT3
Expression
1.0
0.5
0.0
*
scr
siSOCS3
SOCS3
Expression
1.5
1.0
0.5
0.0
**
scr
siSOCS3
E
IL6
hyIL6
H+ produced μmoles
0.6
0.4
0.2
0
0
8
16
Time [h]

**Figure S7. Mechanism, related to Figure 4, 5 and 6.** A: Quantification of western blots corresponding to figures (from left to right) 4C, 4F and 4G. B: MIA PaCa-2 and PANC-1 cells were treated with napabucasin 0.5 M for 4h. Effect of STAT inhibition was confirmed by pSTAT immunoblotting in MIA PaCa-2 cells. Note that PANC1 cells do not respond to napabucasin as these cells lack the enzyme required to produce the biologically active form of drug. PANC1 cells are therefore a useful control to distinguish specific actions of the drug via STAT, from non-specific actions. Acute treatment with napabucasin (0.5-2 M) (red) reduced fermentative rate in MIA PaCa-2 cells and transiently increased oxygen consumption, while having no effect on PANC-1 cells. Plots show mean effect of three biological repeats. Yellow lines correspond to cell-free controls. C. MIA PaCa-2 xenograft growth curve. Female 12-week athymic Nude Crl:NU(NCr)-Foxn1nu mice received sub-cutanous injections with MIA PaCa-2 cells. Each mouse was injected with 2 million cells suspended in 100 µL of a 1:1 mixture of Matrigel and serum-free medium on the left flank. Mice were weighed and the tumors measured 3 times a week. Plot shows mean tumor volume from 5 animals ± SEM. Related to figure 5C. D: Quantification of western blot corresponding to Figure 6E. E: Effect of IL6 (150 ng/mL) and HyIL6 (100 ng/mL) on fermentative rate (N=4) in cells without regular medium replacement. Treatment given 24 h prior to measurements.

Full length blots corresponding to figures:

4C

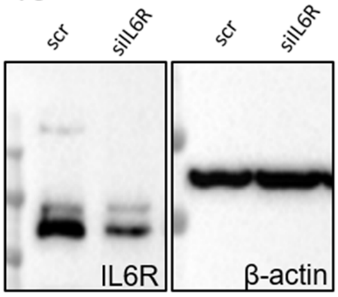


4F

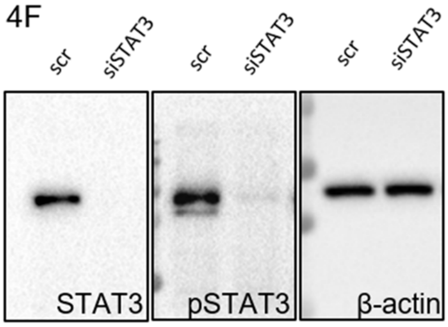


4G

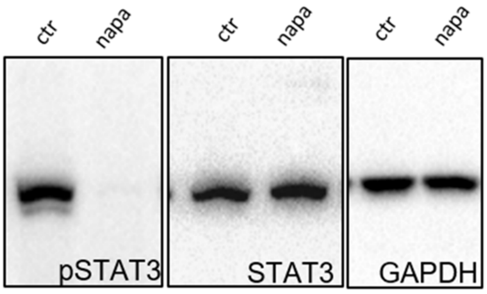


6C

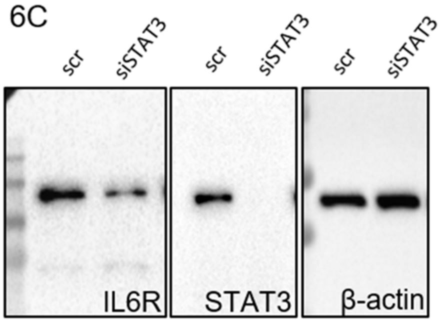


6D

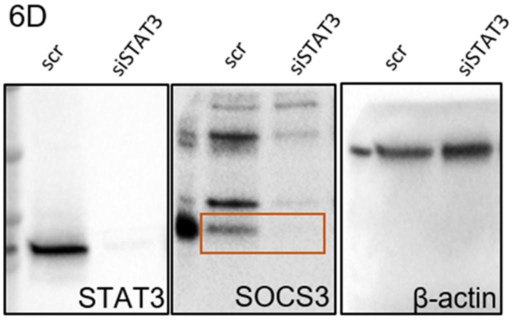


6E

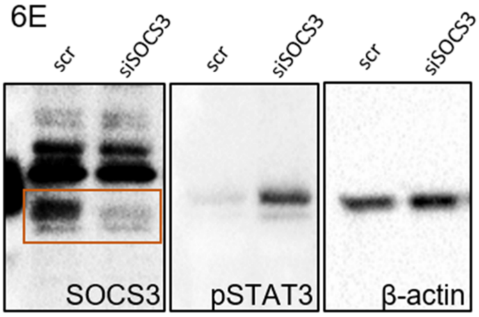


6H

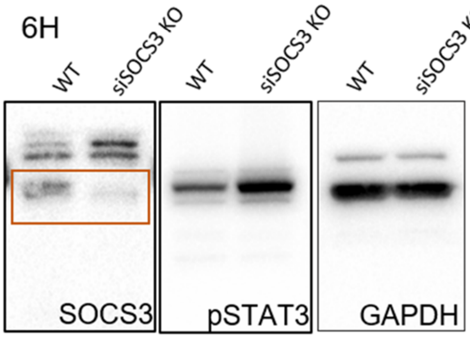


S7B

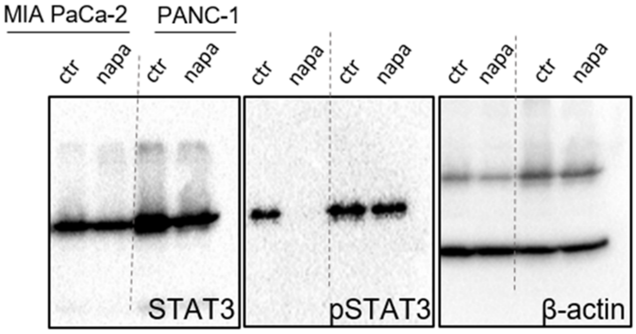

Supplement: Document S1. Table S1 and Figures S1–S7 [file mmc1.pdf]
